# Achieving efficient photodynamic therapy under both normoxia and hypoxia using cyclometalated Ru(ii) photosensitizer through type I photochemical process†
†Electronic supplementary information (ESI) available: Details of general experimental information, X-ray crystallography analysis, CCDC deposition numbers, CV, NMR and MS spectra. CCDC 1509023. For ESI and crystallographic data in CIF or other electronic format see DOI: 10.1039/c7sc03765a


**DOI:** 10.1039/c7sc03765a

**Published:** 2017-10-31

**Authors:** Zhuang Lv, Huanjie Wei, Qing Li, Xianlong Su, Shujuan Liu, Kenneth Yin Zhang, Wen Lv, Qiang Zhao, Xianghong Li, Wei Huang

**Affiliations:** a Key Laboratory for Organic Electronics and Information Displays , Institute of Advanced Materials (IAM) , Jiangsu National Synergetic Innovation Center for Advanced Materials (SICAM) , Nanjing University of Posts and Telecommunications (NUPT) , Nanjing 210023 , P. R. China . Email: iamqzhao@njupt.edu.cn ; Email: wei-huang@njtech.edu.cn; b Key Laboratory of Catalysis and Materials of the State Ethnic Commission & Ministry of Education , South-Central University for Nationalities (SCUEC) , Wuhan 430074 , Hubei Province , P. R. China . Email: lixhchem@mail.scuec.edu.cn; c Shaanxi Institute of Flexible Electronics (SIFE) , Northwestern Polytechnical University (NPU) , Xi’an 710072 , Shaanxi , China

## Abstract

Photodynamic therapy (PDT) through the generation of singlet oxygen utilizing photosensitizers (PSs) is significantly limited under hypoxic conditions in solid tumors. So it is meaningful to develop effective PSs which can maintain excellent therapeutic effects under hypoxia. Here we reported a coumarin-modified cyclometalated Ru(ii) photosensitizer (**Ru2**), which exhibits lower oxidation potential and stronger absorption in the visible region than the coumarin-free counterpart. The evaluation of the PDT effect was performed under both normoxia and hypoxia. The results showed that **Ru2** has a better therapeutic effect than the coumarin-free counterpart in *in vitro* experiments. Especially under hypoxia, **Ru2** still retained an excellent PDT effect, which can be attributed to the direct charge transfer between the excited PS and an adjacent substrate through a type I photochemical process, forming highly-oxidative hydroxyl radicals to damage tumor cells. The anti-tumor activity of **Ru2** was further proven to be effective in tumor-bearing mice, and tumor growth was inhibited remarkably under PDT treatment.

## Abstract 2

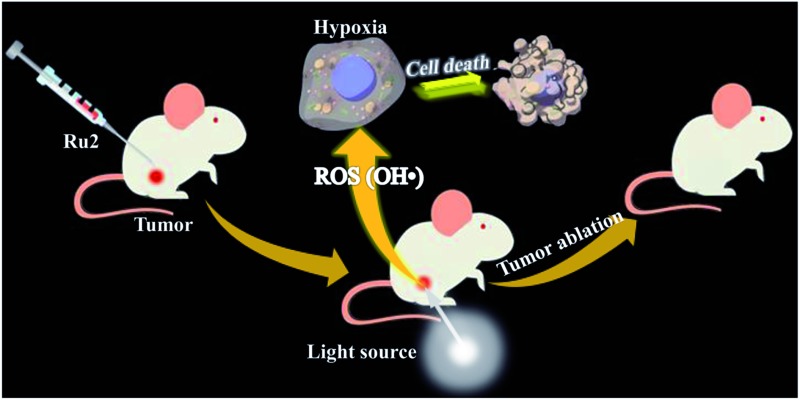
A type I Ru(ii) photosensitizer retained an excellent PDT effect under hypoxia through the formation of highly-oxidative hydroxyl radicals under light irradiation.

## Introduction

Cancer has become one of the most common life-threatening diseases with rising incidence and mortality. Therefore, it is very essential to develop effective methods for cancer therapy. At present, many modalities have been employed for cancer therapy including chemotherapy, surgical therapy, radiation therapy and so on. The main defects of traditional therapies are the non-specific biodistribution of drugs or surgical trauma, which lead to severe side effects to healthy organs. Photodynamic therapy is a special form of therapy to eliminate harmful cells or unhealthy tissues by combining non-toxic photosensitizers with light of an appropriate wavelength. Upon light irradiation, reactive oxygen species (ROS) are generated and then destroy the biomolecules and kill unwanted cells.^1^–^3^ Based on these characteristics, PDT has several distinct advantages compared with other treatments. The photosensitizers often exhibit low dark cytotoxicity and become highly cytotoxic under light irradiation. The size of the treatment region depends on the light irradiation area, which ensures a low side effect to the adjacent healthy tissues. Owing to its minimum invasiveness and high selectivity, photodynamic therapy has attracted tremendous attention for the treatment of many diseases including cancers.^4^–^7^

In general, the mechanism of PDT is based on energy transfer from the excited photosensitizers (PSs) to molecular oxygen for generating singlet oxygen (^1^O_2_), which can instantaneously damage biomolecules to initiate cell death (type II PDT).^8^,^9^ Molecular oxygen is indispensable to type II PDT and the generation of ^1^O_2_ is affected by the surrounding oxygen concentration. Hence, the therapeutic effect of type II PDT is highly dependent on the level of oxygen content and is only initiated under well-oxygenated conditions. However, the native microenvironment in solid tumors is hypoxic due to rapid tumor growth and insufficient oxygen supply.^10^–^12^ Moreover, fast oxygen consumption during PDT further aggravates the hypoxic condition and restricts the therapeutic effect, which has become a major obstacle for PDT.^13^,^14^ In another PDT approach, charge transfer occurs between the excited PS and adjacent substrates, forming a reactive radical ion to damage biomolecules (type I PDT).^15^ This type of PDT can work well under hypoxic conditions and break through the limitation of hypoxia in the type II PDT process.^16^ It is considered that an ideal photosensitizer for type I should have low oxidation potential and good electron-donating ability.^17^ However, most of the reported PSs are assigned to type II PDT, and cannot meet the requirement for type I PDT due to their weak electron-donating ability under excited states. So it is very significant to develop suitable type I PSs possessing excellent therapeutic effects under both normoxia and hypoxia.

Ru(ii) complexes have a wide range of applications in tremendous fields such as solar cells, photocatalysis and biomedicine.^18^–^24^ Especially, they are widely used PSs for PDT because of their excellent excited-state properties and biocompatibility. Most of the reported Ru(ii)-based PSs are based on polypyridyl ligands and exhibit excellent PDT effects *via* the type II process.^25^–^27^ For example, Chao *et al.* reported a series of Ru(ii)-polypyridyl complex based type II photosensitizers exhibiting excellent PDT effects.^25^,^26^ However, cyclometalated Ru(ii) complexes, which have been extensively applied in dye-sensitizer solar cells,^18^ attracted less attention in the biomedical field because of their relatively short-lived triplet excited states undergoing efficient nonradiative decay. Although the following quenched photoluminescence and abated metal-dominated oxidation potentials have restricted the employment of C⁁N cyclometalated Ru(ii) complexes in photoluminescence applications, the electron-donating cyclometalated C⁁N ligands can elevate the energy level of the dπ(Ru) orbital and make the oxidation potential cathodic shift, providing a possibility for type I PSs by electron transfer.^17^

**Scheme 1:**
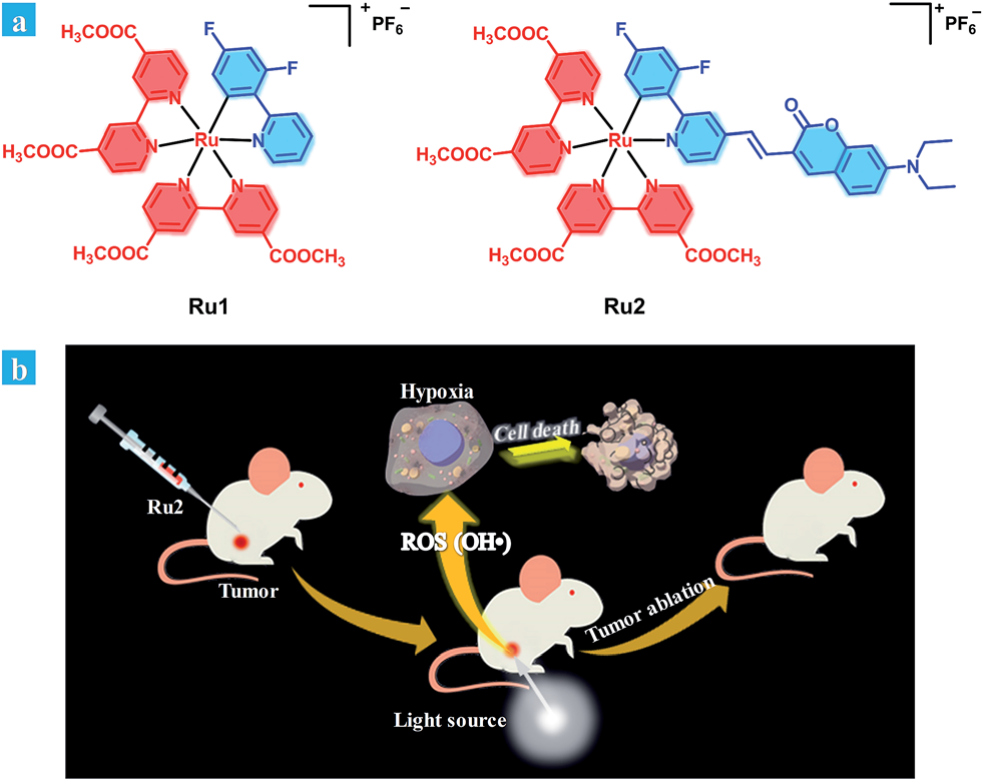
(a) Chemical structures of **Ru1** and **Ru2**. (b) Schematic illustration of **Ru2** used as an efficient type I photosensitizer for photodynamic therapy.

Herein, we provide an effective design strategy to develop type I PSs based on cyclometalated Ru(ii) complexes, and two Ru(ii) complexes (**Ru1** and **Ru2**, Scheme 1a) were designed and synthesized. For **Ru1**, 2,4-difluorophenyl pyridine was used as a cyclometalated ligand. To enhance the light-absorption ability and modulate the energy level of the cyclometalated Ru(ii) complex, a coumarin moiety, which is an excellent chromophore due to its electron-donating and light-harvesting abilities,^28^,^29^ was further introduced into a cyclometalated ligand through a carbon–carbon double bond to obtain complex **Ru2**.

Both the photophysical and electrochemical properties of **Ru1** and **Ru2** were investigated by spectroscopy and cyclic voltammetry. Their reactive oxygen species (ROS) generation abilities were determined and compared with those of a polypyridyl Ru(ii) complex Ru(bpy)_3_^2+^ (a representative type II PS, bpy is the abbreviation of 2,2′-bipyridine). The toxicity of the two cyclometalated Ru(ii) complexes was evaluated by MTT (3-(4,5-dimethylthiazol-2-yl)-2,5-diphenyltetrazolium bromide) assay under dark and light irradiation conditions. As the therapeutic effect could be influenced by oxygen concentration, the evaluation of PDT effects was performed under both normoxia and hypoxia. The results showed that **Ru2** has a better therapeutic effect than **Ru1**. Especially under hypoxia, **Ru2** still retained an excellent PDT effect. Furthermore, the PDT effect of **Ru2** was verified by *in vivo* experiments in tumor-bearing mice.

## Results and discussion

### Synthesis and characterizations

**Scheme 2:**
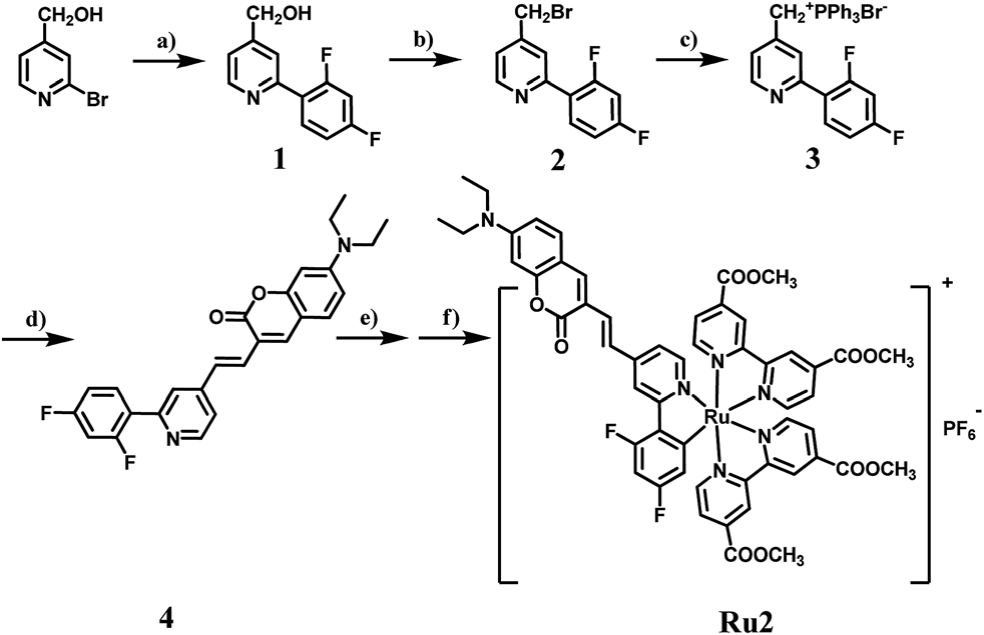
The synthetic route of complex **Ru2**. (a) 2,4-Difluorophenylboronic acid, Pd(PPh_3_)_4_, Na_2_CO_3_, toluene, reflux, 12 h; (b) 48% HBr, reflux, 6 h; (c) PPh_3_, toluene, reflux, 5 h; (d) 7-diethylaminocoumarin-3-aldehyde, K_2_CO_3_, THF, 0 °C; (e) [Ru(cycme)Cl_2_]_2_, CH_3_CN, 50 °C, 24 h; (f) 4,4′-dimethylester-2,2′-bipyridine, CH_3_OH, reflux, 5 h.

The synthetic routes of the C⁁N ligands and cyclometalated Ru(ii) complexes are shown in Scheme 2 and the compounds have been characterized by ^1^H NMR, ^13^C NMR and MS spectra. The intermediate **1** was synthesized according to the classic Suzuki coupling reaction between 4-hydroxymethyl-2-bromopyridine and 2,4-difluorophenylboronic acid. The intermediates **2** and **3** were obtained by a halogenation reaction and nucleophile substitution reaction without chromatography purification, respectively. The coumarin unit was successfully introduced into the C⁁N ligand **4** by a Witting reaction between the intermediate **3** and 7-diethylaminocoumarin-3-aldehyde. The structure of **4** was well characterized by ^1^H NMR, ^13^C NMR and MS spectra and single-crystal X-ray diffraction. The single crystal of **4** was obtained by the recrystallization technique using acetonitrile and dichloromethane as solvents. The details of the crystallographic parameters and ORTEP presentation of **4** are shown in Table S2 and Fig. S1,† respectively. As shown in Fig. S1,† it can be confirmed that the coumarin has been fused to the C⁁N ligand successfully. Finally, **Ru1** and **Ru2** were synthesized using [Ru(cycme)Cl_2_]_2_ and corresponding C⁁N ligands through the two steps method. The acetonitrile–Ru(ii)–C⁁N complexes were obtained in the first step and then the acetonitrile ligands were replaced by 4,4′-dimethylester-2,2′-bipyridine to form cyclometalated Ru(ii) complexes in the second step. The ^1^H NMR spectrum of **Ru2** is shown in Fig. S35,† and the characteristic signals of –N(CH_2_CH_3_)_2_ and –OCH_3_ can be found at 3.44 (4H, –NCH_2_–), 1.13 (6H, –CH_3_) and 3.95 (12H, –OCH_3_). The NMR spectra of **Ru1** and **Ru2** show high signal resolution, indicating that the purity of cyclometalated Ru(ii) complexes is suitable for biological applications.

### Photophysical and redox properties

**Fig. 1:**
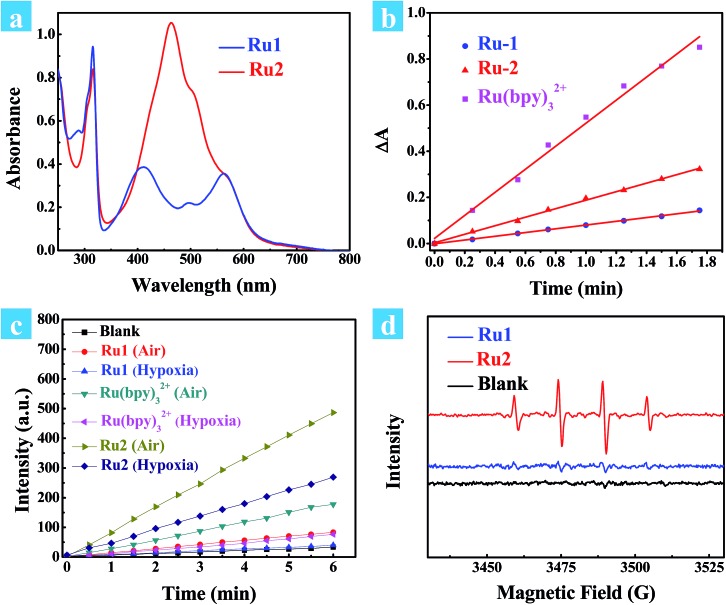
(a) Ground-state absorption spectra of **Ru1** and **Ru2** in CH_3_CN at room temperature. (b) Change in absorbance of DPBF at 410 nm against irradiation time in the presence of Ru(bpy)_3_^2+^, **Ru1** or **Ru2** in methanol. The time interval was 0.25 min. The light source is a xenon lamp with a 475 ± 20 nm output (10 mW cm^–2^). (c) DCF emission intensity at 530 nm as a function of light irradiation time in aqueous solution in the presence of Ru(bpy)_3_^2+^, **Ru1** or **Ru2**. The concentration of the complex is 0.5 μM. (d) EPR signals obtained upon irradiation (400–800 nm, 50 mW cm^–2^) for 5 min of air-saturated PBS solutions of 100 mM DMPO and 10 μM Ru(ii) complexes.

Absorption spectra of cyclometalated Ru(ii) complexes were measured in CH_3_CN at room temperature (Fig. 1a). For **Ru1** and **Ru2**, the sharp and narrow absorption bands in the region of 270–330 nm were assigned to ππ* transitions centered on the N⁁N and C⁁N ligands, and another two low-energy bands between 350–750 nm were assigned to charge transfer (CT) transitions. The introduction of a coumarin chromophore into the C⁁N ligand caused an obvious absorption enhancement in the range of 450–550 nm. Different from polypyridyl-based Ru(ii) complexes, the cyclometalated complexes **Ru1** and **Ru2** are non-emissive at room temperature. However, weak emission bands at 750 nm can be observed at 77 K. The highest occupied molecular orbital (HOMO) and the lowest unoccupied molecular orbital (LUMO) distributions of the cyclometalated Ru(ii) complex were investigated by density functional theory (DFT) calculation. As shown in Fig. S2,† the HOMO of **Ru2** was located on the cyclometalated ligand, and LUMO was on the metal center and N⁁N ligands, while those of **Ru1** were both located on the ruthenium and N⁁N ligand. The redox properties of cyclometalated Ru(ii) complexes were investigated by cyclic voltammetry (Fig. S5 and S6†). **Ru2** shows a reversible oxidation wave at 0.33 V, while **Ru1** and Ru(bpy)_3_^2+^ show oxidation waves at 0.59 V and 0.78 V, respectively (*E*_1/2_*versus* Fc/Fc^+^). Thus, the energy levels of both the HOMO and LUMO can be obtained from the corresponding redox potentials as shown in Scheme S1.† The cyclometalated complexes have a significantly elevated HOMO level compared to Ru(bpy)_3_^2+^. Especially for **Ru2**, the introduction of the coumarin unit into the C⁁N ligand leads to the highest HOMO energy level, which may promote its electron transfer ability to the substrates.

### ROS detection

Next, singlet oxygen generation quantum yields (*Φ*_Δ_) of **Ru1** and **Ru2** were investigated. 1,3-Diphenylisobenzofuran (DPBF) was used as the singlet oxygen scavenger and Ru(bpy)_3_^2+^ as a standard sensitizer (*Φ*_Δ_ = 0.73 in methanol) to measure the relative singlet oxygen quantum yields of **Ru1** and **Ru2**.^30^,^31^ The mixture of Ru(ii) complexes and DPBF was irradiated with 475 nm light (10 mW cm^–2^) for a time interval between 0 to 1.75 min. As shown in Fig. S9,† a decrease in absorbance for DPBF at 410 nm was monitored. The linear relation of absorption change (*A*_0_ – *A*_*n*_) against irradiation time is shown in Fig. 1b, and the slopes of **Ru1**, **Ru2** and Ru(bpy)_3_^2+^ were 0.081, 0.184 and 0.499, respectively. The quantum yields of singlet oxygen were calculated as 0.14 for **Ru1** and 0.16 for **Ru2**. Cyclometalated Ru(ii) complexes have much lower *Φ*_Δ_ than the reference sensitizer Ru(bpy)_3_^2+^. This implies that the energy transfer process is mainly dominated by nonradiative deactivation rather than oxygen sensitization.

To verify the total ROS generating abilities of the cyclometalated Ru(ii) complexes, a ROS probe 2,7-dichloriflurescin diacetate (DCFH-DA) was used for semi-quantitative analysis with Ru(bpy)_3_^2+^ as a reference.^32^,^33^ Thus, the generation of ROS in aqueous solution was investigated. After conversion of DCFH-DA into 2,7-dichloriflurescin (DCFH), DCFH can be further transformed into highly fluorescent 2,7-dichlorofluorescein (DCF) in the presence of ROS. An evident increase in emission for DCF was monitored at 530 nm (Fig. S10 and S11†). As shown in Fig. 1c, an approximate linear relation between the fluorescence intensity of DCF at 530 nm and irradiation time was observed for **Ru1**, **Ru2** and Ru(bpy)_3_^2+^ under both air and 5% O_2_ atmospheres, separately. **Ru2** has a maximum slope in the ROS generation experiments, especially under 5% O_2_ concentration, indicating that it could produce more ROS under light irradiation than **Ru1** and Ru(bpy)_3_^2+^. Though **Ru2** has a relatively lower singlet oxygen quantum yield than Ru(bpy)_3_^2+^, a higher level of total ROS generation of **Ru2** was observed under both normoxia and hypoxia. It is especially important that total ROS produced by **Ru2** also remained 3.6 times higher than that produced by Ru(bpy)_3_^2+^ and 6.7 times higher than that produced by **Ru1** under hypoxic conditions. These results indicate that some other reactive intermediates are produced by light irradiation.

Furthermore, the electron paramagnetic resonance (EPR) spin-trapping technique was used to demonstrate the ROS generation induced by cyclometalated Ru(ii) complexes with 5,5-dimethyl-1-pyrroline-N-oxide (DMPO) as a spin-trapping agent.^34^,^35^ After irradiation of the air-saturated PBS/CH_3_CN (9 : 1, v/v) solutions of **Ru1** and **Ru2** (10 μM) and DMPO (100 mM), a quartet of signals with a relative intensity of 1 : 2 : 2 : 1 from the DMPO-OH adduct were acquired after irradiation, suggesting that hydroxyl radicals (OH˙) were formed. The EPR signal intensity of **Ru2** was much stronger than that of **Ru1**, which is consistent with the results of ROS generation. In this way, the generation of hydroxyl radicals under light irradiation indicates that **Ru2** can work as a type I photosensitizer, making it applicable for PDT under hypoxic conditions.^36^,^37^ Then, the therapeutic effect of **Ru2** was evaluated under both normoxia and hypoxia.

### *In vitro* PDT effects under both normoxia and hypoxia

**Fig. 2:**
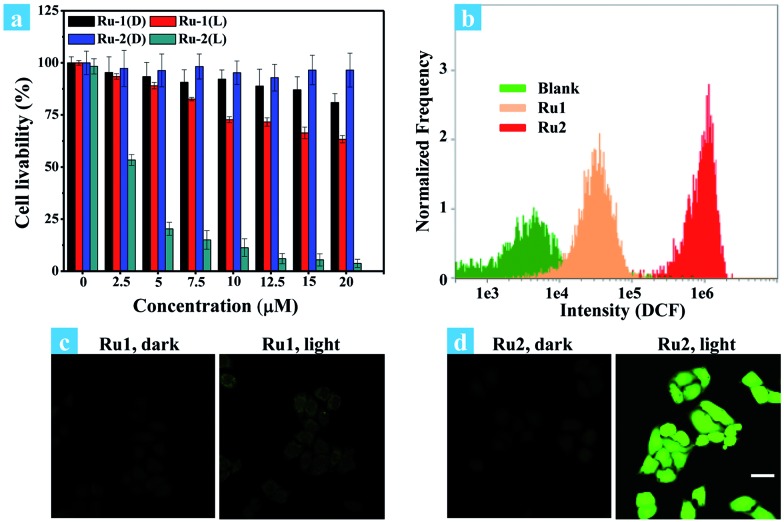
(a) Dose–dependent curves for cell viability of HeLa cells treated with **Ru1** and **Ru2** using a typical MTT assay under light irradiation (L) or in the dark (D). Cells were irradiated with white light (400–800 nm, 30 mW cm^–2^, 10 min). (b) Flow cytometric assay of fluorescence intensity of DCF, cells incubated with Ru(ii) complexes (5 μM) were treated with light irradiation (35 mW cm^–2^). (c and d) Confocal fluorescence images of ROS generation in cells incubated with Ru(ii) complexes (5 μM), cells were treated with light irradiation (400–800 nm, 35 mW cm^–2^) for 10 min. The images share the same scale bar of 50 μm.

The potential application of **Ru2** for PDT has been evaluated and compared with **Ru1** under normoxia. In the first step, the phototoxicity of **Ru1** and **Ru2** to HeLa cells was performed by the typical colorimetric cell viability MTT experiments under normoxia and the results are shown in Fig. 2a. IC_50_ (half maximal inhibitory concentration of cell viability) values were obtained after 4 h of incubation following light irradiation (white light, 400–800 nm, 30 mW cm^–2^, 10 min). As shown in Fig. 2a, the cell viability was 3.1% at a concentration of 20 μM. Otherwise, **Ru2** showed low cytotoxic effect up to a concentration of 20 μM under dark conditions. The results implied that **Ru2** exhibited phototoxicity to HeLa cells in the level of micro molar concentration. Furthermore, 48 h MTT assay has been also performed (Fig. S8†), and the results revealed that **Ru2** exhibits low dark cytotoxicity to HeLa cells under the experimental conditions in this work (5 μM). Then, ROS generation within the cells was proved by confocal fluorescence imaging and flow cytometry assay. After incubation of HeLa cells with Ru(ii) complexes and DCFH-DA, the emission channel of 505–565 nm was collected under different conditions.^38^Fig. 2c and d showed representative confocal images of DCF fluorescence after white light (400–800 nm) irradiation (35 mW cm^–2^, 10 min) in Ru(ii) complex loaded HeLa cells. After irradiation, the bright emission of DCF appeared in the **Ru2** group, suggesting a high level of ROS generation in the cells. In contrast, weak background fluorescence of DCF was observed in the **Ru1** group (Fig. 2c) and control group of **Ru2** (Fig. 2d). These results suggest that intracellular ROS generation can be induced by **Ru2** under light irradiation, which was more efficient than that of **Ru1**.

Fig. 2b shows the effect of irradiation on ROS generation in cancer cells, which was detected by flow cytometry. For comparison, blank and **Ru1** groups showed low intensity of DCF emission while significant fluorescence enhancement was observed in the **Ru2** group. The average signal intensity of DCF in the **Ru2** group was 116 times higher than that in the blank group. The fluorescence and bright field images of HeLa cells under different treatment conditions were also obtained from flow cytometry (Fig. S14†). The results of emission intensity and cell morphology were in line with confocal fluorescence imaging shown in Fig. 2c and d. As shown in Fig. S14,† the HeLa cell exhibits good cell morphology in the control group, indicating that **Ru2** shows low dark cytotoxicity to HeLa cells. After light irradiation, the cell morphology has been seriously damaged, indicating high phototoxicity of **Ru2** to the cells. These results demonstrated that the generation of ROS induced by **Ru2** was directly correlative to cell death.

**Fig. 3:**
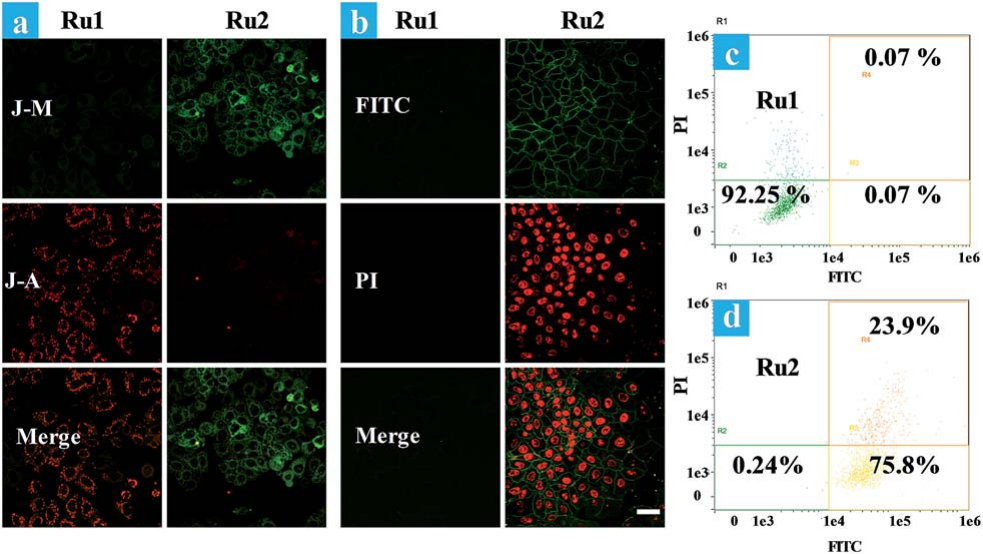
(a) Fluorescence images of JC-1 stained HeLa cells. Ru(ii) complex (5 μM) loaded HeLa cells were treated by light irradiation (400–800 nm, 50 mW cm^–2^, 15 min). Cells were viewed in the red channel for J-aggregates (*λ*_ex_ = 515 nm, *λ*_em_ = 580–640 nm) and the green channel for JC-1 monomers (*λ*_ex_ = 515 nm, *λ*_em_ = 530–560 nm). J-A and J-M stand for the J-aggregates and J-monomers. (b) Time-dependent confocal fluorescence images of annexin V-FITC/PI stained cells at 4.5 h after light irradiation, the cells were incubated with Ru(ii) complexes (5 μM) under normoxia. (c and d) Flow cytometric assay of cell death under normoxia. The images share the same scale bar of 50 μm.

The effects of **Ru1** and **Ru2** on the mitochondrial membrane potential (MMP) change of HeLa cells have also been studied under different irradiation conditions. JC-1 is a dual-emission potential-sensitive probe that can be used to monitor mitochondrial membrane potential.^39^,^40^ At higher potentials in living cells, JC-1 forms red-fluorescent “J-aggregates”, while a green-fluorescent monomer can be formed at low membrane potential in apoptotic cells. The **Ru1** group (Fig. 3a) and control group of **Ru2** (Fig. S15†) exhibited similar results showing intense red emission localized in the mitochondria and weak green emission in the cytoplasm. Conversely, treatment of HeLa cells by light irradiation caused the decrease of red emission in the mitochondria and the increase of green emission in the cytoplasm (Fig. 3a). The observation of cell morphology from bright field also indicated cell death after light irradiation. This observation is in agreement with the green-emission monomer form of JC-1 in the apoptotic cells.

Furthermore, time-lapse fluorescence imaging was performed for real-time monitoring of the progress of photoinduced cell apoptosis.^38^**Ru1** and **Ru2** induced cell apoptosis was investigated and monitored for a long time *via* CLSM under normoxia (Fig. 3b). Signals of annexin FTIC/PI have not been observed in the blank and **Ru1** groups at 4.5 h and similar results were also obtained from the control group of **Ru2** (Fig. S16–S19†). These results indicated that **Ru2** exhibited low toxicity for long-term culture and observation under dark conditions. After **Ru2** loaded HeLa cells were treated by a white light (400–800 nm, 35 mW cm^–2^) under normoxia, green fluorescence of annexin V-FITC appeared at 0.5 h after light irradiation, indicating the early stage apoptosis of HeLa cells. Red fluorescence with apoptotic characteristics was observed at 1.0 h after irradiation (Fig. S20†), indicating that **Ru2** could induce HeLa cell apoptosis rapidly under light irradiation.

To further demonstrate the therapeutic effect of **Ru1** and **Ru2** by quantitative analysis, experiments of cell apoptosis were performed. In flow cytometry assay, annexin V-TITC–/PI– (viable cells), annexin V-TITC+/PI– (early apoptotic cells), and annexin V-TITC+/PI+ (late apoptotic cells) were used to distinguish the different stages of cell death.^38^,^41^ After HeLa cells were incubated with 5 μM Ru(ii) complexes for 2 h and then irradiated with white light (400–800 nm) at a dose of 35 mW cm^–2^, the cells were stained with annexin V-FITC/PI. As shown in Fig. 3d, the percentage of **Ru2** induced apoptotic cells under normoxia was measured to be 75.8% for early apoptosis and 23.9% for late apoptosis. As a contrast, the percentages of apoptotic cells in the **Ru1** group and control group of **Ru2** were 7.75% (Fig. 3c) and 4.87%, respectively (Fig. S21†). The results indicated the low dark cytotoxicity and good therapeutic effect of **Ru2** under normoxia. All the above experiments showed that **Ru2** could be a promising photosensitizer for PDT because of its low dark toxicity and high phototoxicity to HeLa cells under normoxia, while **Ru1** was ineffective under the same conditions.

**Fig. 4:**
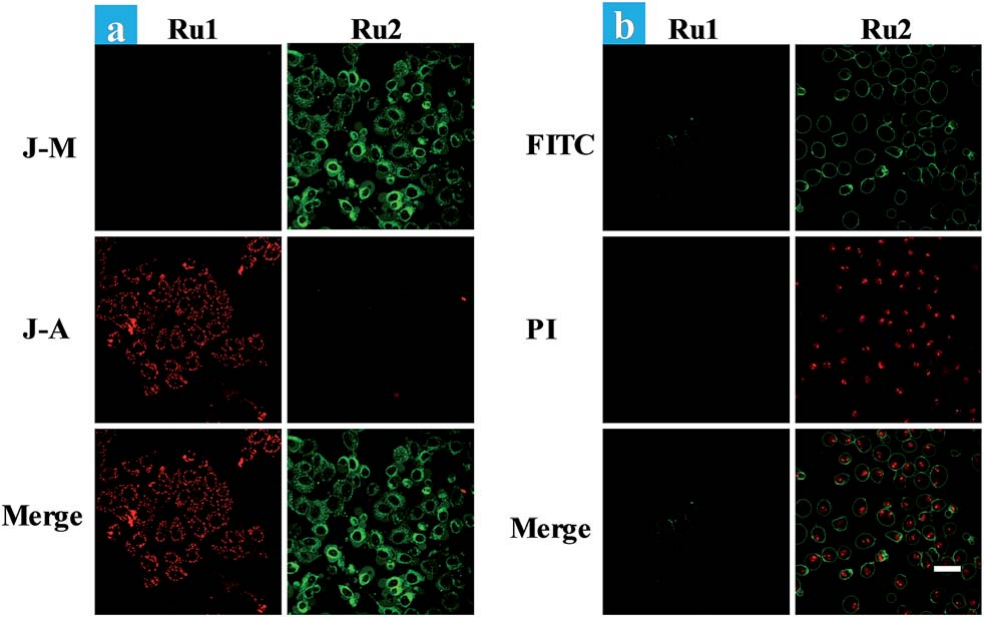
(a) Fluorescence images of JC-1 stained HeLa cells. Ru(ii) complex (5 μM) loaded HeLa cells were treated by light irradiation (400–800 nm, 50 mW cm^–2^, 15 min) under hypoxia. Cells were viewed in the red channel for J-aggregates (*λ*_ex_ = 515 nm, *λ*_em_ = 580–640 nm) and the green channel for JC-1 monomers (*λ*_ex_ = 515 nm, *λ*_em_ = 530–560 nm). (b) Time-dependent confocal fluorescence images of annexin V-FITC/PI stained cells with different treatments. The cells were incubated under 5% O_2_ and irradiated by white light (400–800 nm, 35 mW cm^–2^) with a xenon lamp for 10 min. The images were taken from **Ru2** and **Ru1** mediated PDT at 4.5 h after light irradiation. The images share the same scale bar of 50 μm.

Cancer cells in tumors always retain a hypoxic atmosphere, severely limiting the therapeutic effect of type II PDT. Hence, as a type I photosensitizer of **Ru2**, the generation of hydroxyl radicals was favorable for PDT under hypoxic conditions. The changes of MMP on **Ru1** and **Ru2** loaded HeLa cells have been observed under hypoxic conditions (Fig. 4a). The fluorescence in **Ru1** and **Ru2** groups was transferred from red to green, indicating that cell apoptosis has occurred in the **Ru2** group. Furthermore, the progress of photoinduced cell apoptosis under hypoxic conditions (5% O_2_) was observed by time-lapse fluorescence imaging (Fig. S24–S28†). After HeLa cells were incubated with 5 μM Ru(ii) complexes for 2 h and then irradiated with white light (400–800 nm) for 10 min at a power of 35 mW cm^–2^ under 5% O_2_, the cells can be stained with annexin V-FITC/PI. **Ru2** loaded HeLa cells exhibited features of cell death at 1.5 h after light irradiation (Fig. S28†), while **Ru1** loaded cells showed no signal of apoptosis even at 4.5 h (Fig. 4b). The results indicated that the process of apoptosis in the **Ru2** group is significantly faster than that of the **Ru1** groups under hypoxia.

**Fig. 5:**
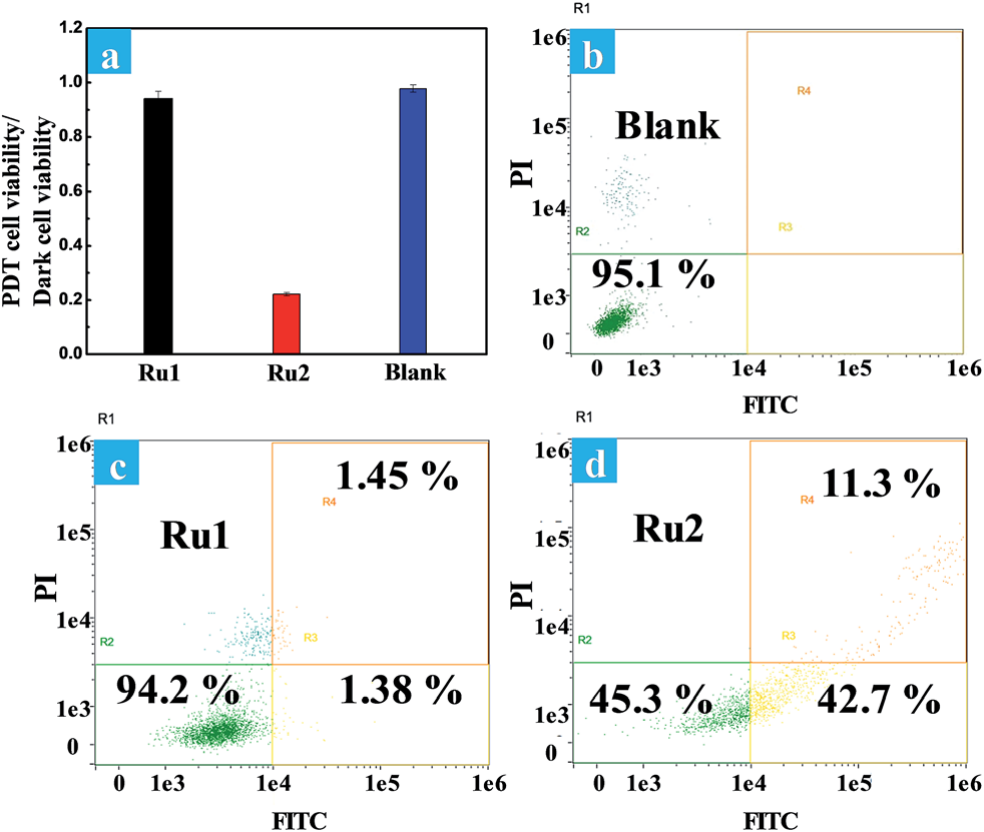
(a) The ratios of PDT cell viability to dark cell viability under hypoxia. Cells were irradiated with white light (400–800 nm, 30 mW cm^–2^, 10 min). Flow cytometric assay of cell death induced by (b) control cells, (c) **Ru1** and (d) **Ru2** (5 μM) mediated PDT under hypoxia.

The phototoxicity of **Ru1** and **Ru2** to HeLa cells was compared by the typical colorimetric cell viability MTT experiments under hypoxia. The ratios of PDT cell viability to dark cell viability were obtained as follows: 0.94 for **Ru1** and 0.22 for **Ru2** (Fig. 5a). The results implied that **Ru2** exhibited significant phototoxicity to HeLa cells under hypoxia, which is much higher than that of **Ru1**. The quantitative analysis of the therapeutic effect of Ru(ii) complexes under hypoxia was performed by flow cytometry. Percentages of apoptotic cells of each cyclometalated Ru(ii) complex under hypoxia have been quantified as 2.83% for **Ru1** and 54.0% for **Ru2** (Fig. 5c and d). The above quantitative analysis of cell apoptosis under hypoxic conditions exhibited that **Ru2** was more effective than **Ru1**. These results obtained from the above experiments revealed that **Ru2** could be a promising photosensitizer for PDT under hypoxic conditions.

Based on the above experimental results, the proposed mechanism of **Ru2** for PDT was described as follows (Schemes 1b and S2†). Hydroxyl radicals were generally produced by electrons transferred from the excited PS to substrates under light irradiation.^42^ Firstly, electrons could be transferred from the excited states of the PS to adjacent substrates (PS or biomolecular), which then induced the formation of radical cations and anions. The hydroxyl radicals were generated from two steps. One was electron transfer from the hydroxyl to radical cations (PS˙+) and the other was electron transfer to molecular oxygen by radical anions (PS˙– or substrate˙–). In the latter step, the Harber–Weiss reaction could be initiated by the formation of a superoxide radical. In this reaction, the molecular oxygen was utilized as a recyclable agent. So the ROS generation by the PS under hypoxia still remains at a suitable level. Then hydroxyl radicals induced the biomolecular inactivation and further caused cell death. As illustrated in Scheme S2,† **Ru2** can induce cancer cell death *via* the synergistic effect of these two steps under both normoxia and hypoxia, and the generation of hydroxyl radicals ensured the therapeutic effect especially under hypoxic conditions.

### *In vivo* photodynamic therapeutic efficacy of **Ru2**

**Fig. 6:**
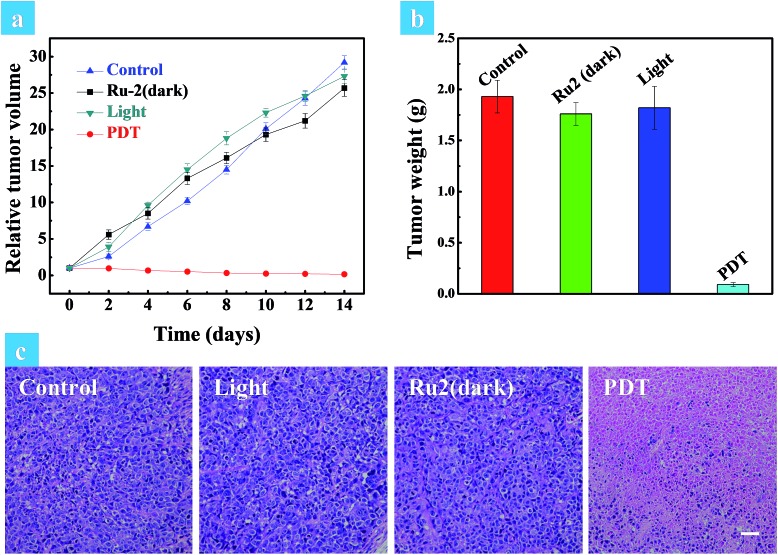
(a) Relative tumor volume of different groups after various treatments. (b) Tumor weights of different groups after 14 days of treatment. The mice were sacrificed after 14 days and the tumors were obtained. (c) H&E stained tumor slices of different groups. The images share the same scale bar of 100 μm.

The above *in vitro* experimental results revealed that **Ru2** has excellent therapeutic effects under hypoxia. So *in vivo* experiments were then performed to verify the therapeutic effect of **Ru2**. Solid tumors are generally exposed to a hypoxic microenvironment. Hence HeLa tumor-bearing mice were chosen as a model. All mice were divided into four groups randomly (four mice in each group: control group, dark group, light group and PDT group) when the tumor volume reached experimental requirements (∼50 mm^3^). **Ru2** was injected intratumorally into the tumor bearing mice at a dose of 5 mg kg^–1^ and PDT was initiated by a xenon lamp (250 mW cm^–2^, 15 min) at 15 min post injection. The relative tumor volumes were measured every two days to observe the therapeutic effect. As shown in Fig. S29,† the tumor volume of the control group, dark group and light group was increased dramatically from 0 days to 14 days, while the tumor growth of the PDT group was remarkably inhibited in the same period. The tumor growth curves of the different groups are shown in Fig. 6a. At last the tumors were removed and then weighed. The results showed that the tumor weight in the PDT group was much smaller than that of other groups (Fig. 6b). These results revealed that **Ru2** can effectively inhibit tumor growth under light irradiation and has little dark toxicity to tumor tissues. Finally the mice were sacrificed after 14 days and then the tumors and normal organs were obtained. Hematoxylin and eosin (H&E) staining of tumor sections after various treatments was performed to investigate the therapeutic effect by histological analysis. After H&E staining, the nucleus can be stained to violet-blue and the cytoplasm can be stained to red. The H&E staining of the tumor section of the dark group shows negligible necrosis, revealing that **Ru2** has little dark toxicity to tumor cells (Fig. 6b). As a contrast, the section of the PDT group showed serious cell damage, revealing that **Ru2** has excellent antitumor efficacy under light irradiation.

**Fig. 7:**
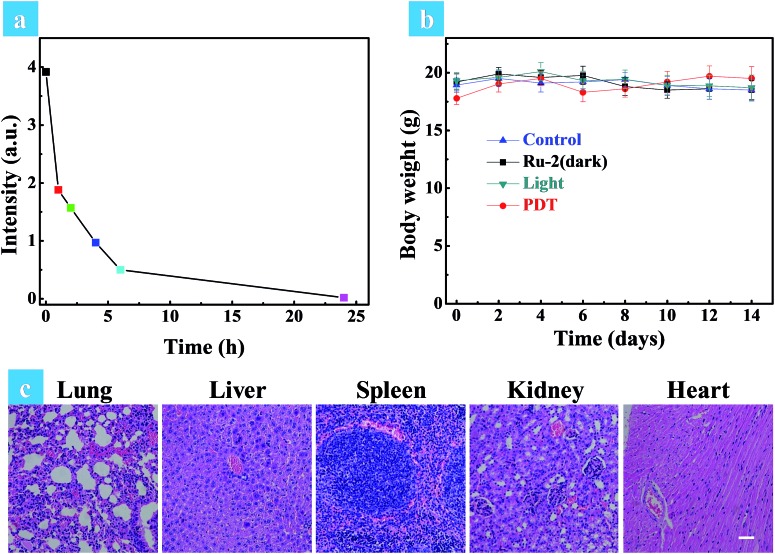
(a) The quantitative fluorescence signal intensities of Ru(bpy)_3_(PF6)_2_ at different time intervals in the solid tumor. (b) Time-dependent body weight curves of different groups after various treatments. (c) H&E stained tissue slices of normal organs (lung, liver, spleen, kidney, and heart) in the PDT group after 14 days of treatment. The images share the same scale bar of 100 μm.

A small animal *in vivo* imaging system was then applied to estimate the clearing time of **Ru2** in a solid tumor. Due to the non-emissive property of **Ru2**, the red emissive Ru(bpy)_3_(PF_6_)_2_ as an analog was injected intratumorally into the tumor bearing mice to investigate the residence time in the tumor. As shown in Fig. S30,† the maximum signal intensity in the tumor can be observed at 15 min post injection. The signal intensity gradually decreased as time went on and finally disappeared at 24 h post injection, inferring that **Ru2** can also be removed quickly from the tumor. To investigate the side effects of **Ru2** to tumor bearing mice, the changes of body weight and pathological histology were observed as indicators.^43^ The body weights of the mice in different groups were recorded every two days. As shown in Fig. 7b, the body weights were not significantly changed in the four groups after 14 days of treatment. The slight weight loss of the PDT group in the early stage was perhaps induced by tumor ablation. The normal organs (heart, liver, spleen, lung, and kidney) of all groups were also obtained after 14 days of treatment and then stained by H&E. The images of the stained slides of the organs were acquired by microscopy and are shown in Fig. 7c and S31.† No obvious pathological abnormalities were found in the organ sections of all groups by histological analysis.^44^ These results indicated that all treatment conditions have no side effects to mice after 14 days, especially for the **Ru2** treated groups. Therefore, it can be concluded that **Ru2** is not toxic to normal organs and has no cumulative effect in the body.

## Conclusions

In summary, we have developed a coumarin-modified cyclometalated Ru(ii) complex **Ru2** as a type I photosensitizer for hypoxic PDT. **Ru2** exhibits an excellent PDT effect under both normoxia and hypoxia, which is much better than that of coumarin-free **Ru1**. It has been demonstrated that the formation of a hydroxyl radical under both normoxia and hypoxia plays a key role in inducing cell death. So the therapeutic effect of the photosensitizer could be less affected by the oxygen concentration. The *in vivo* experiments also demonstrated that **Ru2** has an excellent PDT effect in a model of an endogenous hypoxic solid tumor. On the other side, **Ru2** shows negligible dark toxicity to the cancer cells and normal organs at the experimental concentration. All of these results revealed the promising application of **Ru2** for PDT, especially under hypoxia conditions. We think that this work is meaningful for designing and developing highly effective type I PDT agents based on cyclometalated Ru(ii) complexes by rationally tuning their charge transfer ability.

## Experimental section

### Materials

All solvents were obtained from commercial sources and used without further purification. 2,4-Difluorophenylboronic acid, Pd(PPh_3_)_4_ and [Ru(cycme)Cl_2_]_2_ were purchased from Across and used without further purification. 2,4-Difluorophenylpyridine,^45^ 4-hydroxymethyl-2-bromopyridine,^46^ 7-diethylaminocoumarin-3-aldehyde^47^ and 4,4′-dimethylester-2,2′-bipyridine^48^ were prepared and purified as described in the literature, and were then characterized by NMR.

### Instruments

NMR spectra were obtained using a Bruker Avance-400 spectrometer. Mass spectra were obtained using an AVANCE III and AB Sciex MALDI-TOF-TOF spectrometer. Single-crystal X-ray diffraction was measured on a Bruker SMART 1000 CCD diffractometer with Mo Kα radiation (*λ* = 0.071073 nm) using the w-scan mode. Data were corrected for absorption using the SADABS program, and solution and refinement of the structure were performed using the SHELX-97 software package.^49^ The UV-visible absorption spectra were obtained using a Shimadzu UV-3600 UV-VIS-NIR spectrophotometer. Photoluminescence spectra were obtained using an Edinburgh FL 920 spectrophotometer equipped with a temperature controller. For the spectra and cell imaging experiments, flow counters (HORIBA STEC, SEC-E40JS, 60 SCCM) or oxygen concentration-changeable multi-gas incubators (Thermo Scientific, SERIES II WATER JACKET CO_2_ Incubator, Model: 3131, S/N: 112620-1988) were used to control the oxygen concentrations. A xenon lamp (CEL-HXF 300, *P* = 300 W) was used as the light source during the PDT process. Confocal luminescence imaging was obtained using an Olympus FV1000 confocal laser scanning microscope equipped with a 40 immersion objective lens. Flow cytometry experiments were conducted on flow sight.

### Synthesis of **1**, **2**, **3** and **4**

See the ESI.†

### Synthesis and characterization of **Ru1**

A suspension of [Ru(cycme)Cl_2_]_2_ (0.075 g, 0.12 mmol), triethylamine (0.05 mL), 2,4-difluorophenylpyridine (0.042 g, 0.22 mmol) and KPF_6_ (0.081 g, 0.44 mmol) in 15 mL of acetonitrile was stirred at 50 °C for 24 h under nitrogen atmosphere. The orange solid was obtained by the solvent evaporation method and then added into methanol solution (10 mL) containing 4,4′-dimethylester-2,2′-bipyridine (0.10 g, 0.37 mmol). After the solution was refluxed for 5 h under nitrogen atmosphere, the solvent was removed by evaporation. The crude compound was purified by column chromatography on silica gel using CH_3_CN/CH_2_Cl_2_ as the eluent to afford the product as a black-red solid. Yield: 0.045 g (47%). ^1^H NMR (DMSO-d_6_, 400 MHz), *d* (ppm): 9.25 (s, 1H), 9.18 (s, 1H), 9.15 (s, 2H), 8.27 (d, *J* = 8.8 Hz, 2H), 8.17 (d, *J* = 5.6 Hz, 1H), 7.99 (d, *J* = 5.6 Hz, 1H), 7.93–7.86 (m, 6H), 7.75 (dd, *J* = 5.6 Hz, *J* = 1.2 Hz, 1H), 7.54 (d, *J* = 5.6 Hz, 1H), 7.12 (t, *J* = 6.6 Hz, 1H), 6.72–6.66 (m, 1H), 5.94 (d, *J* = 7.6 Hz, 1H), 3.96 (t, *J* = 6.0 Hz, 12H). ^13^C NMR (DMSO-d_6_, 400–800 MHz), *d* (ppm): 197.19, 164.74, 164.67, 164.66, 157.76, 157.15, 156.74, 155.39, 154.92, 151.51, 151.25, 150.89, 150.64, 138.10, 137.88, 136.66, 135.63, 135.52, 135.50, 128.22, 127.14, 126.52, 126.42, 126.34, 123.84, 123.73, 123.57, 123.46, 123.23, 118.52, 99.99, 53.69, 53.63, 53.55. MS (MALDI-TOF, CHCA): *m*/*z* calculated 835.75 (M – PF_6_^–^), found: 835.67.

### Synthesis and characterization of **Ru2**

According to a similar synthetic procedure for **Ru1**, complex **Ru2** was prepared using compound **4** (0.096 g, 0.22 mmol) as the C⁁N-ligand and a black-red solid was finally obtained. Yield: 0.052 g (20%). ^1^H NMR (DMSO-d_6_, 400 MHz), (ppm): 9.26 (s, 1H), 9.19 (s, 1H), 9.16 (s, 2H), 8.25 (s, 1H), 8.20 (t, *J* = 5.6 Hz, 2H), 8.01 (t, *J* = 5.8 Hz, 2H), 7.94 (q, *J* = 5.3 Hz, 4H), 7.76 (d, *J* = 5.8 Hz, 1H), 7.54–7.43 (m, 3H), 7.36 (d, *J* = 16.0 Hz, 1H), 7.26 (d, *J* = 5.8 Hz, 1H), 6.76 (m, 2H), 6.55 (s, 1H), 5.76 (d, *J* = 7.7 Hz, 1H), 3.96 (t, 12H), 3.46 (d, *J* = 6.2 Hz, 4H), 1.13 (t, *J* = 6.6 Hz, 6H). ^13^C NMR (DMSO-d_6_, 100 MHz), (ppm): 197.11, 164.74, 164.66, 163.02, 160.48, 157.76, 157.13, 156.73, 156.25, 155.42, 154.91, 151.65, 151.47, 151.40, 150.66, 150.56, 146.22, 142.59, 137.89, 136.65, 135.65, 135.51, 130.48, 130.42, 130.18, 128.22, 127.13, 126.51, 126.43, 126.34, 125.70, 123.84, 123.69, 123.53, 123.43, 120.13, 119.89, 115.05, 110.17, 108.79, 99.99, 96.71, 53.67, 53.59, 53.52, 44.69, 41.98, 12.81. MS (MALDI-TOF, CHCA): *m*/*z* calculated 1077.22 (M – PF_6_^–^), found: 1077.27.

### Cell culture

The HeLa cell lines (human cervical cancer) were obtained from the Institute of Biochemistry and Cell Biology, SIBS, CAS (China). The cells were grown in DMEM (Dulbecco’s modified Eagle’s medium) supplemented with 10% FBS (fetal bovine serum), 100 mg mL^–1^ streptomycin and 100 U mL^–1^ penicillin at 37 °C with 5% CO_2_.

### Singlet oxygen quantum yields (*Φ*_Δ_)

Singlet oxygen quantum yields (*Φ*_Δ_) were detected through monitoring the absorption change of 1,3-diphenylisobenzofuran (DPBF). The air-saturated solution of the photosensitizer containing 50 μM DPBF was prepared under dark conditions and irradiated with a 475 nm xenon lamp in an interval of 0.25 min. Absorption of DPBF was monitored by a UV-Vis spectrophotometer. The *Φ*_Δ_ values were obtained by the relative method using Ru(bpy)_3_^2+^ in methanol (*Φ*_Δ_ = 0.73) as the standard and calculated with eqn (S1).

S1 *Φ*_Δ_(PS) = *Φ*_Δ_(Std)*S*_PS_ × *F*_Std_/(*S*_Std_ × *F*_PS_)

where subscripts PS and Std designate the cyclometalated Ru(ii) complexes and Ru(bpy)_3_^2+^, respectively; *S* is the slope of the plot of the absorbance of DPBF (at 410 nm) against irradiation time; *F* is the correction factor of absorption, which is given by *F* = 1–10^–OD^ (OD means the optical density of Ru(ii) complexes and Ru(bpy)_3_^2+^ at 475 nm).

### ROS detection in aqueous media

To convert DCFH-DA to DCFH, 0.5 mL ethanol solution of DCFH-DA (1 mM) was added to 2 mL aqueous solution of NaOH (0.01 M) and allowed to sit at room temperature for 30 min. The hydrolysate was then neutralized with 10 mL of 25 mM sodium phosphate buffer at pH 7.4, and then stored on ice in the dark. The final concentration of DCDH alkali activated solution was 40 μM.^50^ 10 μL of 10^–4^ M Ru-complex solution was added into 2 mL of the activated DCFH solution. The fluorescence signal of DCF was monitored after the solution was irradiated by white light (400–800 nm) under both air and 5% O_2_ atmosphere. Fluorescence spectra of DCF were recorded in a range of 490–610 nm with the excitation wavelength at 480 nm.

### ROS detection in HeLa cells

HeLa cells were seeded and allowed to incubate overnight. The cells were incubated with Ru(ii) complexes for 2 h and treated with DCFH-DA (10 μM) for 20 min at 37 °C, then irradiated by white light (400–800 nm, 35 mW cm^–2^) with a xenon lamp. The fluorescence intensity of DCF was detected by confocal microscopy and flow cytometry (*λ*_ex_ = 488 nm and *λ*_em_ = 500–540 nm).

### Analysis of mitochondrial membrane potential

HeLa cells were seeded and allowed to incubate overnight. The cells were incubated with Ru(ii) complexes for 2 h at 37 °C with 5% CO_2_ and then irradiated by white light (400–800 nm, 50 mW cm^–2^) with a xenon lamp. After 15 min, the cells were treated with JC-1 (5 μg mL^–1^) for 20 min at 37 °C. The fluorescence intensity of JC-1 was measured by confocal microscopy. Cells were viewed on a confocal microscope in the red channel for J-aggregates (*λ*_ex_ = 515 nm, *λ*_em_ = 580–640 nm) and the green channel for JC-1 monomers (*λ*_ex_ = 515 nm, *λ*_em_ = 530–560 nm).

### Annexin V-FITC/PI assay

HeLa cells were seeded and allowed to incubate overnight. Then the cells were incubated with Ru(ii) complexes for 2 h at 37 °C with 5% CO_2_. The cells were further incubated for an additional 1 h under 5% O_2_ for the measurement of apoptosis under hypoxia and then irradiated by white light (400–800 nm, 35 mW cm^–2^) with a xenon lamp. After 10 min, the cells were stained with annexin V-FITC and PI according to the protocol (KeyGEN, China). Confocal microscopy and flow cytometry were used to measure the fluorescence intensity of the cells (annexin V-FITC, *λ*_ex_ = 488 nm, *λ*_em_ = 500–560 nm; PI, *λ*_ex_ = 488 nm, *λ*_em_ = 600–680 nm).

### Confocal luminescence imaging

Confocal luminescence imaging was carried out using an Olympus IX81 confocal laser scanning microscope equipped with a 40 immersion objective lens. A semiconductor laser at 488 nm was provided for excitation of the HeLa cells. The HeLa cells were incubated with the Ru(ii) complexes (5 μM) for 2 h at 37 °C. Then the cells were incubated with an annexin V-FITC Apoptosis Detection Kit or ROS kit as per the manufacturer’s protocols after treatment under different conditions. Then the cells were put into the Live Cell Imaging System (OLYMPUS, Xcellence) for confocal luminescence imaging immediately.

### Cell viability assay by MTT

HeLa cells were harvested and subcultured in 96-well plates for 24 h before the experiment at a density of 4–7 × 10^4^ cells per well. Ru(ii) complexes with varying concentrations were respectively added into the wells followed by further culture for additional 2 h. Then the fresh cell growth medium (150 μL) was added to the wells after the culture media containing Ru(ii) complexes were discarded. The cells were further incubated for an additional 1 h under 5% O_2_ for the measurements under hypoxia. PDT treatment was performed using a white light (400–800 nm) obtained by a xenon lamp with a power density of 30 mW cm^–2^ for 10 min. After irradiation, the cells were allowed to continue incubation for 4 h. MTT (1 mg mL^–1^, 150 μL per well) was added to the wells after the culture media were discarded and then incubated at 37 °C for another 4 h. The liquid in the wells were removed, and DMSO (150 μL) was added to dissolve the produced formazan. The plates were shaken for 10 min and the absorbance values of the wells were then read with a microplate reader at a wavelength of 520 nm. The cell viability rate (VR) was obtained with the control group in the absence of Ru(ii) complexes.

### Tumor model

HeLa tumor-bearing mice were provided by Keygenbio Co., Ltd and then fed following the protocol from Nanjing University of Posts and Telecommunications Animal Center.

### *In vivo* antitumor studies

All mice were divided into four groups randomly (control group, dark group, light group and PDT group) and each group had four mice. When the tumors reached a volume of 50 mm^3^, **Ru2** was injected intratumorally into the tumor bearing mice at a dose of 5 mg kg^–1^ and PDT was initiated by a xenon lamp (250 mW cm^–2^, 15 min) at 15 min post injection every 2 days. The relative tumor volumes and body weights were measured every two days. The mice were sacrificed after 14 days of treatment as per the institutional guidelines. Tumors and organs were obtained and then fixed.

### X-ray crystallography data

CCDC number of **4** is ; 1509023.†

## Conflicts of interest

We declared that we have no conflicts of interest to this work.

## Supplementary Material

Supplementary information

Crystal structure data
