# Supplementary material for: Achieving efficient photodynamic therapy under both normoxia and hypoxia using cyclometalated Ru(ii) photosensitizer through type I photochemical process
Source: Chem Sci. 2017 Oct 31;9(2):502–12. doi: 10.1039/c7sc03765a (PMC5868078; doi:10.1039/c7sc03765a)
Supplement: Supplementary file 1 [file SC-009-C7SC03765A-s001.pdf]

Electronic Supplementary Material (ESI) for Chemical Science.
This journal is © The Royal Society of Chemistry 2017

Electronic Supplementary Information

# Achieving efficient photodynamic therapy under both normoxia and hypoxia using cyclometalated Ru(II) photosensitizer through type I photochemical process

Zhuang Lv,[a] Huanjie Wei,[a] Qing Li,[b] Xianlong Su,[b] Shujuan Liu,[a] Kenneth Yin Zhang,[a] Wen Lv,[a] Qiang Zhao,*[a] Xianghong Li*[b] and Wei Huang*[ac]

**Synthesis and characterization of Ru1 and Ru2**

***2-(2,4-Difluorophenyl)pyridyl-4-methanol*** (**1**): To a mixture of 4-hydroxymethyl-2-bromopyridine (3.0 g, 16 mmol), 2,4-difluorophenylboronic acid (2.8 g, 18 mmol) and $Pd(PPh_3)_4$ (0.092 g, 0.80 mmol) in deoxygenated toluene (150 mL), ethanol (15 mL) and the aqueous solution of potassium carbonate (2 M, 30 mL) were added. The resulted mixture was refluxed for 12 h and then cooled to room temperature. After extracted by dichloromethane, solvent removal afforded to a crude product, which was purified by chromatography on silica gel using petroleum/chloroform (3:1, v/v) as eluent. A white powder (2.5 g) was obtained with a yield of 72%. $^1$H NMR (DMSO-$d_6$, 400 MHz): 8.64 (d, *J* = 5.0 Hz, 1 H), 8.02–7.97 (m, 1 H), 7.72 (s, 1 H), 7.41–7.34 (m, 2 H), 7.25–7.21 (m, 1 H), 5.52 (t, *J* = 5.5 Hz, 1 H), 4.62 (d, *J* = 6.0 Hz, 2 H).

***4-(Bromomethyl)-2-(2,4-difluorophenyl)pyridine*** (**2**): The compound **1** (1.8 g, 8.1 mmol) was dissolved in a mixture of 48% HBr (20 mL) and concentrated sulfuric acid (6.7 mL). The resulted solution was refluxed for 6 h and then cooled down to room temperature. Subsequently, 40 mL of ice water was added and the pH of the solution was adjusted to neutral with NaOH solution. The resulted precipitate was filtered, washed with water and air-dried. Then, the product was dissolved in chloroform (40 mL) and filtered. The solution was dried over magnesium sulfate and then evaporated to dryness to give a white powder (2.0 g) with yield of 85%. $^1$H NMR ($CDCl_3$, 400 MHz), $\delta$ (ppm): 8.71 (s, 1 H), 8.04 (d, *J* = 6.5 Hz, 1 H), 7.78 (s, 1 H), 7.32 (s, 1 H), 7.04 (s, 1 H), 6.95 (t, *J* = 9.3 Hz, 1 H), 4.48 (s, 2 H).

***(2-(2,4-Difluorophenyl)-4-methylpyridyl)triphenylphosphonium bromide*** (**3**): $PPh_3$ was added into the toluene solution of **2** and then the mixture was refluxed for 5 h. After cooled to room temperature, the precipitates was obtained and used without further purification for the next Wittig reaction.

***3-(2-(2-(2,4-Difluorophenyl)pyridin-4-yl)vinyl)-7-diethylaminocoumarin*** (**4**): 7-Diethylaminocoumarin-3-aldehyde (0.35 g, 1.4 mmol) was dissolved in 50 mL of THF. After purged with nitrogen for 15 min, the compound **3** (1.1 g, 2.0 mmol) was added. Then the mixture was cooled with ice-water bath, and $K_2CO_3$ (1.38 g, 1 mmol) was added into the solution repeatedly. The mixture was then stirred for 3 h at room temperature. After filtration to remove excess salt, the solvent was then removed by evaporation. The crude compound was purified directly by column chromatography on silica gel using petroleum ether/ethyl acetate (3:2, v/v) as eluent to afford yellow solid (0.29 g, 47%). $^1$H NMR (DMSO-$d_6$, 400 MHz) , $\delta$ (ppm): 8.65 (d, *J* = 5.0 Hz, 1 H), 8.17 (s, 1 H), 8.01 (q, *J* = 8.1 Hz, 1 H), 7.82 (s, 1

H), 7.56–7.39 (m, 5 H), 7.25 (t, *J* = 8.2 Hz, 1 H), 6.76 (t, *J* = 8.1 Hz, 1 H), 6.58 (s, 1 H), 3.47 (q, *J* = 6.8 Hz, 4 H), 1.14 (t, *J* = 6.8 Hz, 6 H). $^{13}C$ NMR (DMSO-$d_6$, 100 MHz), $\delta$ (ppm): 160.50, 156.14, 152.82, 151.47, 150.60, 146.00, 142.42, 132.86, 132.82, 132.72, 130.34, 129.19, 126.57, 121.33, 121.25, 119.93, 115.43, 110.05, 108.75, 105.04, 99.99, 96.74, 44.66, 12.82. MS (EI): m/z calculated 433.17 ($M+H^+$), found: 433.01.

**Octanol/water partition coefficient (log $P_{o/w}$) measurements.**

5 mL of the rutheniu mcomplex in octanol (saturated with PBS) was added to an equal volume of PBS (saturated with octanol), and the mixture was sealed and then stirred 24 h at 298 K to allow partitioning. After centrifuging the sample at 3000 rpm for 10 min, two layers were carefully separated and the Ru concentrations in the two phases were measured by ICP-MS and used to calculate the $[Ru]_o/[Ru]_w$ ratio.

**Table S1.** Photophysical properties of **Ru1** and **Ru2**.

| **Sensitizer** | $\lambda_{abs}$ (nm) ($\varepsilon \times 10^{-4}$, $M^{-1}$ $cm^{-1}$) | | | |
|---|---|---|---|---|
| **Ru1** | 316 (9.41) | 411 (3.86) | 562 (3.56) | |
| **Ru2** | 314 (8.40) | 463 (10.53) | 503 (7.38) | 562 (3.55) |

**Table S2.** Crystal data and structure refinement for **4**.

| | | |
|---|---|---|
| Identification code | **4** | |
| Empirical formula | $C_{26} H_{22} F_2 N_2 O_2$ | |
| Formula weight | 432.46 | |
| Temperature | 296(2) K | |
| Wavelength | 0.71073 Å | |
| Crystal system | Monoclinic | |
| Space group | P2(1)/c | |
| Unit cell dimensions | a = 8.3521(17) ° | $\alpha$= 90° |
| | b = 12.418(2) ° | $\beta$=97.923(4) ° |
| | c = 20.681(4) ° | $\gamma$= 90° |
| Volume | 2124.5(7) $Å^3$ | |
| Z | 4 | |
| Density (calculated) | 1.352 $Mg/m^3$ | |
| Absorption coefficient | 0.098 $mm^{-1}$ | |
| F(000) | 904 | |
| Crystal size | 0.12 × 0.10 × 0.10 $mm^3$ | |
| Theta range for data collection | 1.92 to 29.97° | |
| Index ranges | -11≤ h ≤11, -17≤ k ≤17, -29≤ l ≤25 | |
| Reflections collected | 22259 | |
| Independent reflections | 6107 [$R_{(int)}$ = 0.0485] | |
| Completeness to theta = 29.97° | 98.9 % | |
| Absorption correction | None | |
| Max. and min. transmission | 0.9903 and 0.9883 | |
| Refinement method | Full-matrix least-squares on $F^2$ | |
| Data / restraints / parameters | 6107 / 0 / 292 | |
| Goodness-of-fit on $F^2$ | 0.974 | |
| Final R indices [I>2sigma(I)] | $R_1$ = 0.0488, $wR_2$ = 0.1254 | |
| R indices (all data) | $R_1$ = 0.1150, $wR_2$ = 0.1594 | |
| Extinction coefficient | 0.0014(6) | |
| Largest diff. peak and hole | 0.183 and -0.165 e $Å^{-3}$ | |

**Table S3.** Selected bond lengths [Å] and angles [°] for **4**.

| | | | | | |
|---|---|---|---|---|---|
| F(1)-C(2) | 1.3581(18) | N(1)-C(11) | 1.327(2) | N(2)-C(23) | 1.452(2) |
| C(8)-C(7) | 1.378(2) | C(12)-C(9) | 1.459(2) | C(12)-C(13) | 1.333(2) |
| C(14)-C(15) | 1.449(2) | C(14)-C(16) | 1.357(2) | C(18)-C(17) | 1.388(2) |
| O(1)-C(15) | 1.2032(19) | O(2)-C(15) | 1.385(2) | O(2)-C(18) | 1.3759(19) |
| C(18)-O(2)-C(15) | 122.39(13) | C(11)-N(1)-C(7) | 116. 83(15) | N(2)-C(20)-C(19) | 121.61(17) |
| C(13)-C(12)-C(9) | 125.31(16) | C(12)-C(13)-C(14) | 129.69(17) | C(10)-C(9)-C(12) | 120.06(16) |
| C(3)-C(2)-C(1) | 124.23(15) | N(1)-C(11)-C(10) | 124.56(17) | O(1)-C(15)-O(2) | 114.85(15) |
| O(1)-C(15)-C(14) | 127.21(16) | O(2)-C(15)-C(14) | 117.93(14) | | |

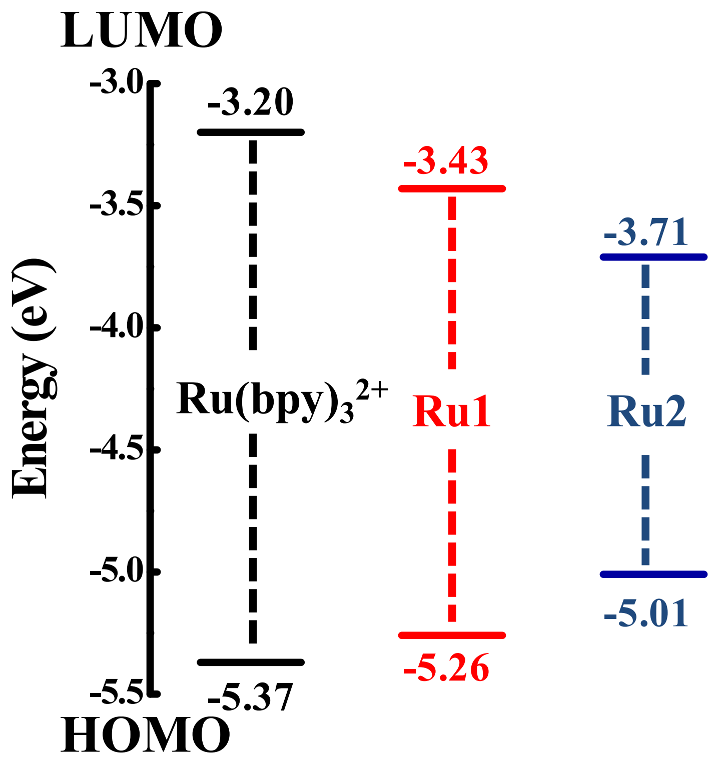


**Scheme S1.** HOMO and LUMO energy levels of **Ru1**, **Ru2** and $Ru(bpy)_3^{2+}$, respectively. The HOMO and LUMO were calculated from following equation (HOMO (LUMO) = − ($E_{onset\text{-}Ru}$ − $E_{onset\text{-}ferrocene}$) − 4.8 eV.), and the onset potentials of Ru-complexes were measured with a ferrocene reference by cyclic voltammetry.

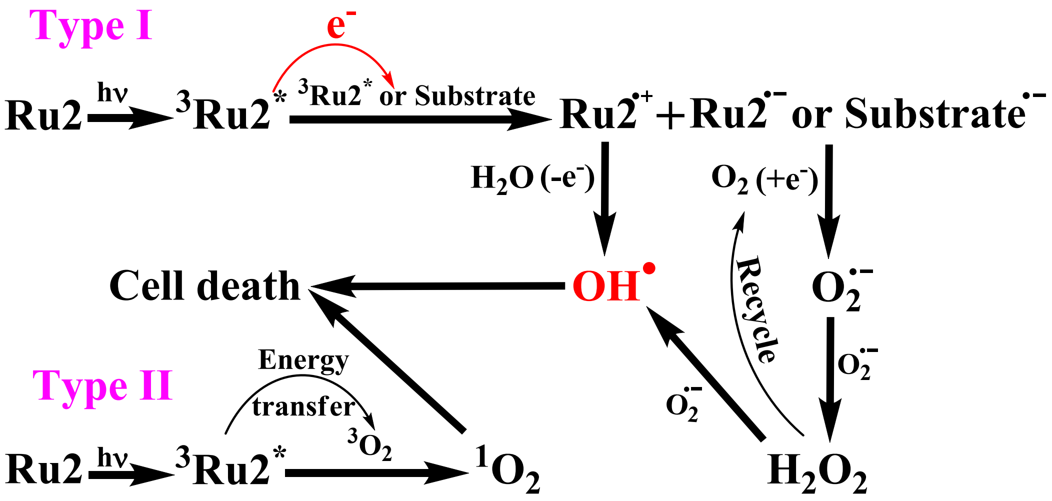


**Scheme S2.** Proposed mechanisms of **Ru2** for PDT.

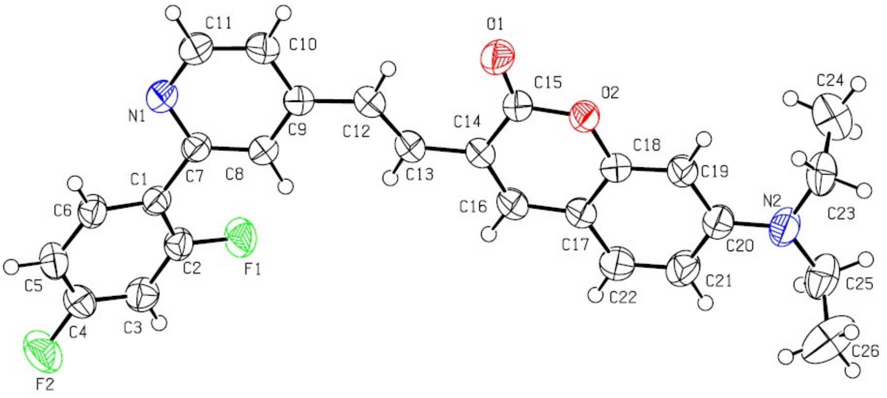


**Figure S1.** ORTEP representation of the X-ray structure of **4**.

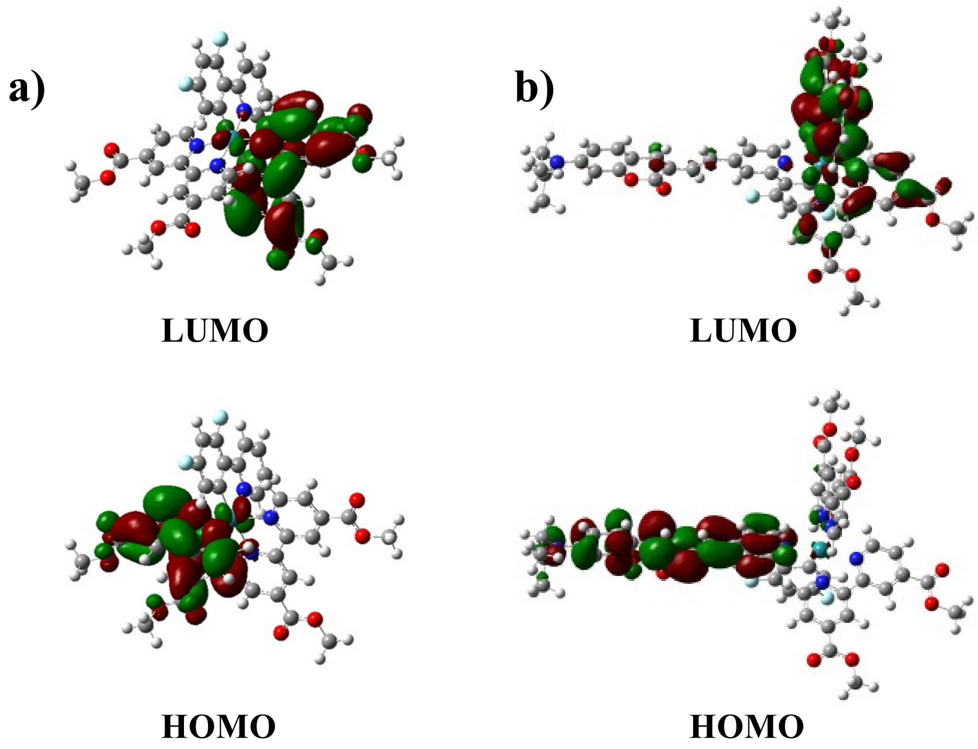


**Figure S2**. HOMO and LUMO distributions of **Ru1** (a) and **Ru2** (b) at $T_1$ state.

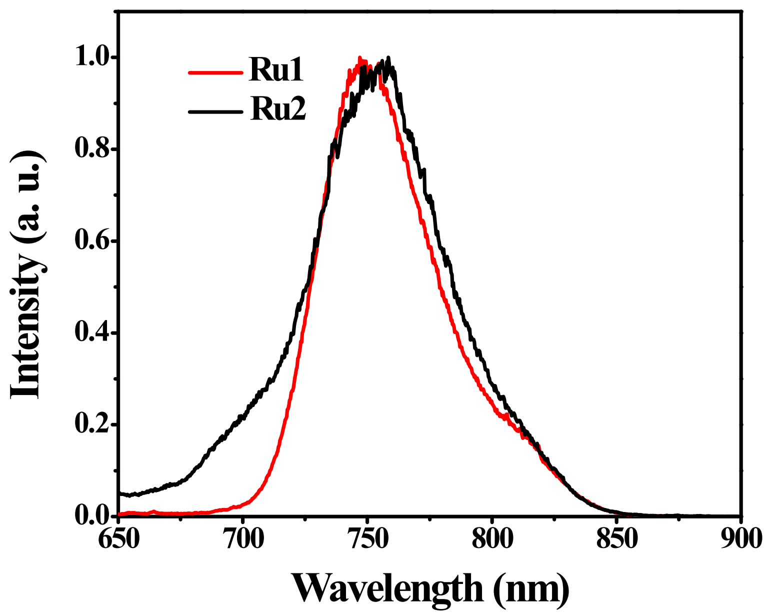


**Figure S3.** Normalized emission spectra of **Ru1** ($\lambda_{ex}$ = 405 nm) and **Ru2** ($\lambda_{ex}$ = 465 nm) in 2-methyltetrahydrofuran at 77 K.

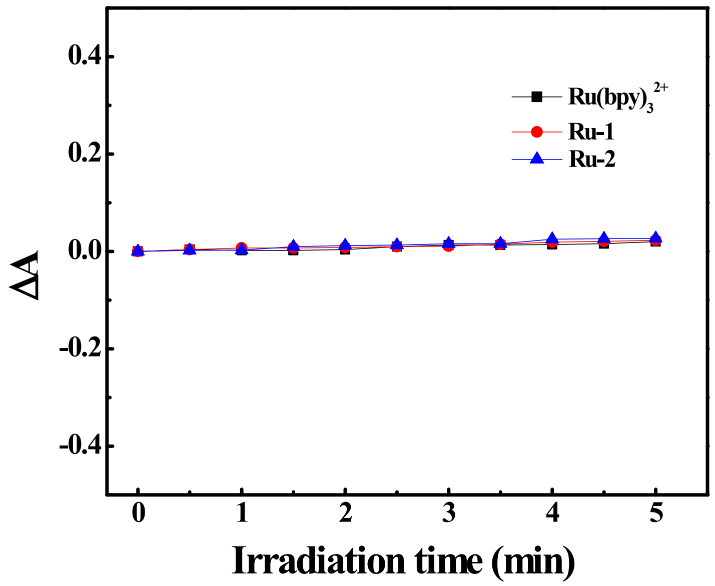


**Figure S4.** Absorption changes of **Ru1**, **Ru2** and $Ru(bpy)_3^{2+}$ in methanol solution under different irradiation time (400–800 nm, 50 mW/cm$^2$) at 25 °C.

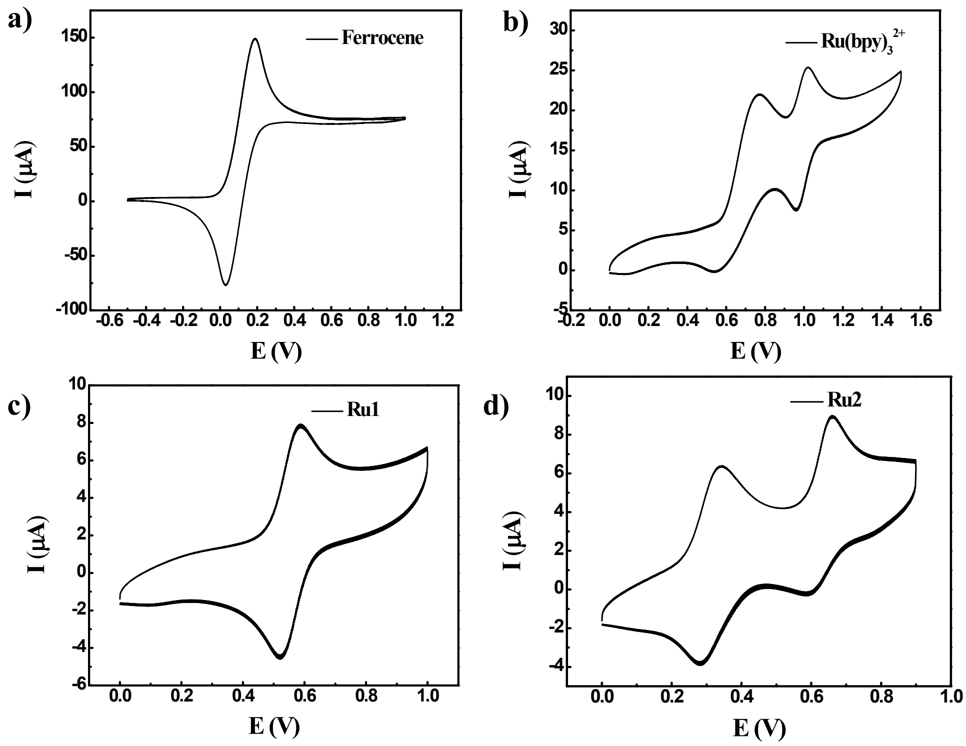


**Figure S5.** Cyclic voltammograms of ferrocene (a), $Ru(bpy)_3^{2+}$ (b), **Ru1** (c) and **Ru2** (d) in $CH_3CN$ with 0.01 M $TBAPF_6$ as the electrolyte at room temperature. Data were collected using the standard three electrode setup with a glassy carbon working electrode, platinum wire counter electrode and $Ag/AgNO_3$ reference electrode.

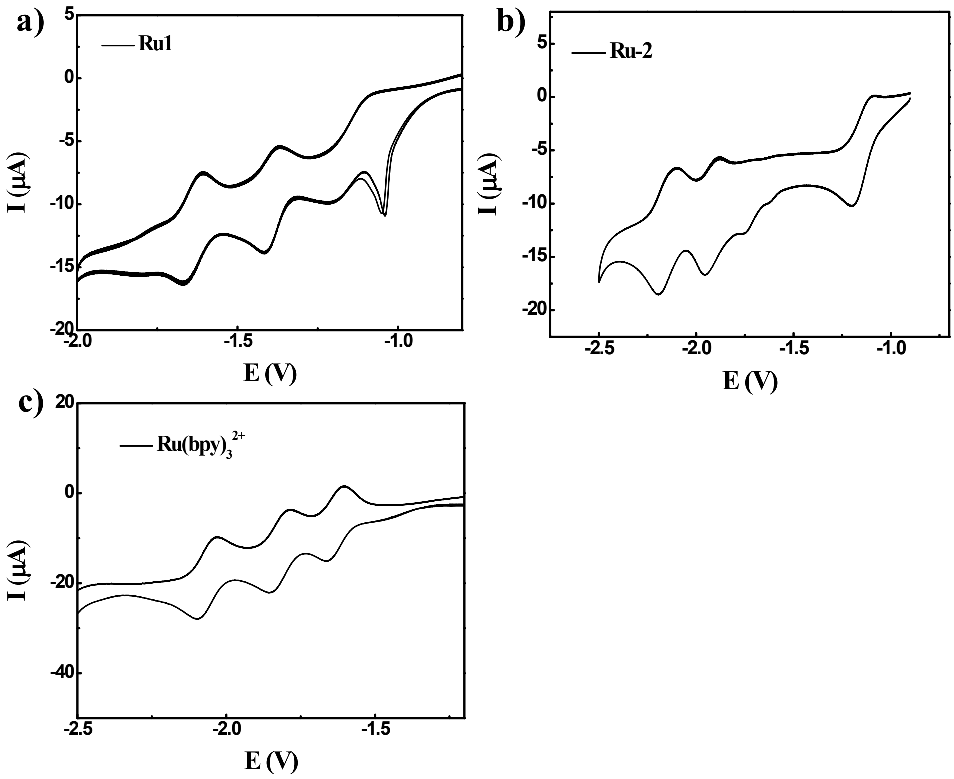


**Figure S6.** Cyclic voltammograms of **Ru1** (a), **Ru2** (b) and $Ru(bpy)_3^{2+}$ (c), in $CH_3CN$ with 0.01 M $TBAPF_6$ as the electrolyte at room temperature. Data were collected using the standard three electrode setup with a glassy carbon working electrode, platinum wire counter electrode and $Ag/AgNO_3$ reference electrode.

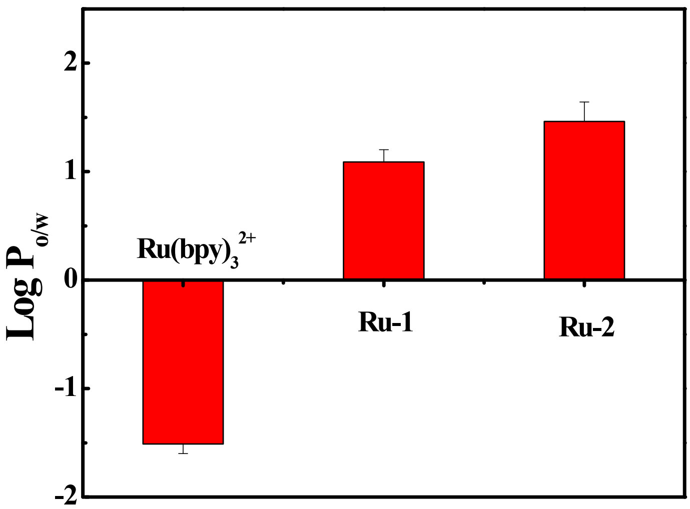


**Figure S7.** Octanol/water partition coefficients of Ru-complexes.

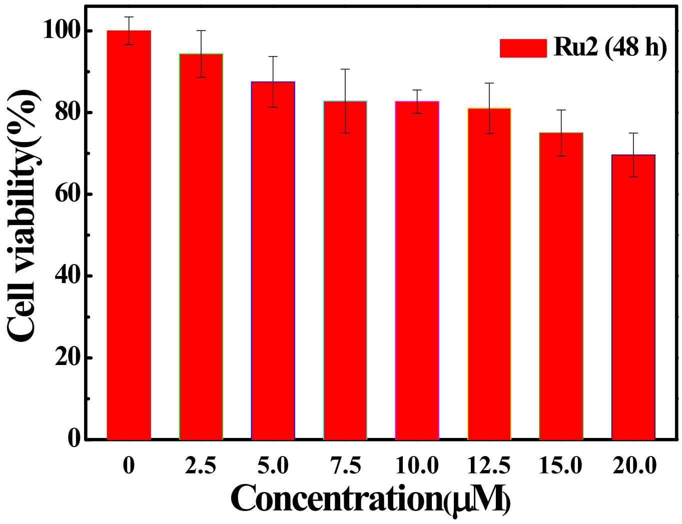


**Figure S8.** Dose-dependent curves for cell viability of Hela cells treated with **Ru2** by using a typical MTT assay under dark condition.

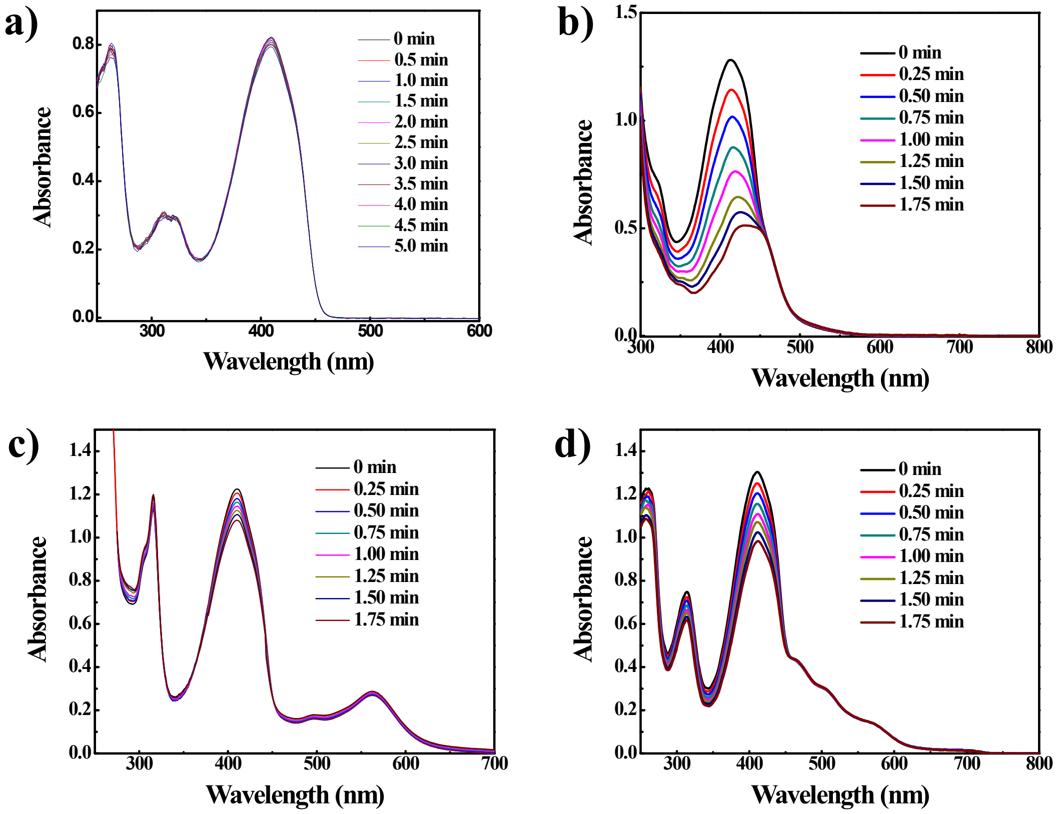


**Figure S9.** Absorption spectra of DPBF at different irradiated time without (a) or in the presence of $Ru(bpy)_3^{2+}$ (b), **Ru1** (c) and **Ru2** (d), respectively. The time interval was 0.5 min of a) and 0.25 min of c-d). The light source is a xenon lamp with a 475 ± 20 nm output (10.0 mW/cm$^2$).

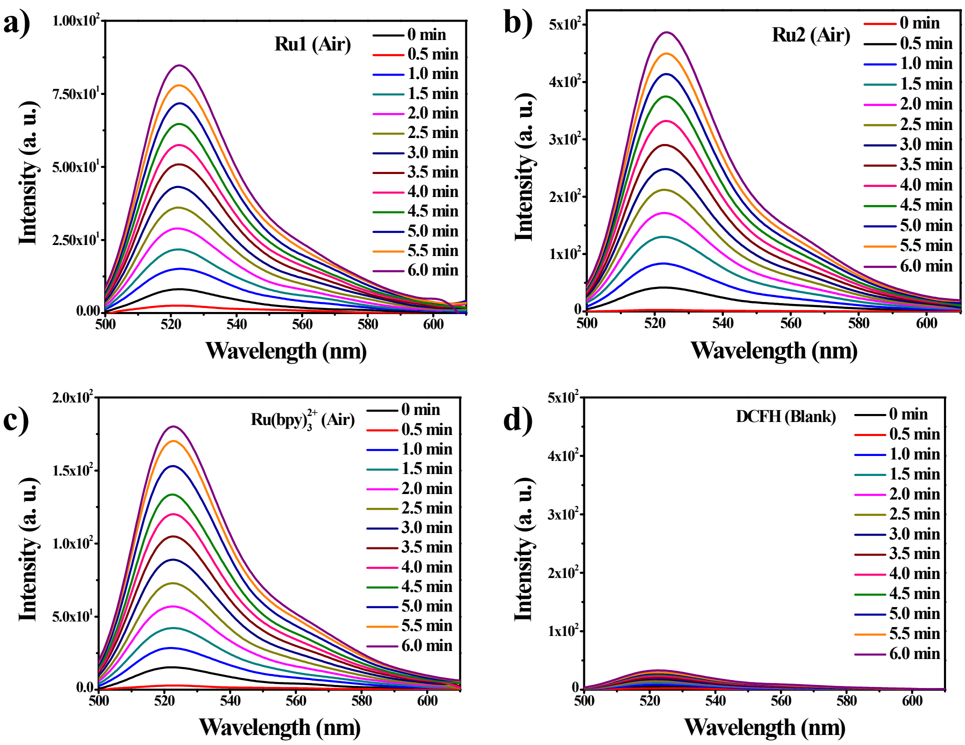


**Figure S10.** a-d) Emission spectra of DCF at different irradiated time in the presence of **Ru1**, **Ru2**, Ru(bpy)$_3^{2+}$ and blank, respectively. The time interval was 0.25 min. The light source is a xenon lamp with a white light (400–800 nm) output (10.0 mW/cm$^2$). All groups were in the air-saturated solutions.

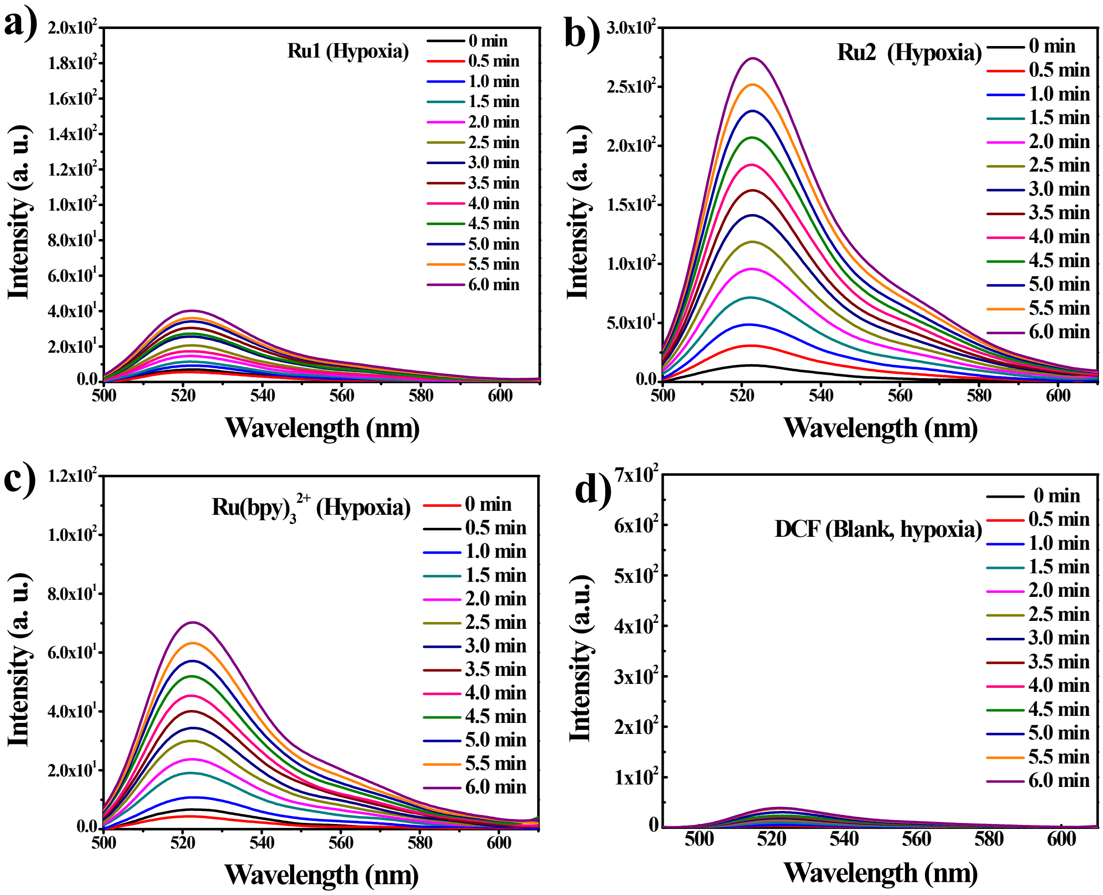


**Figure S11.** a-c) Emission spectra of DCF at different irradiated time in the presence of **Ru1**, **Ru2**, $Ru(bpy)_3^{2+}$ and blank respectively. The time interval was 0.25 min. The light source is a xenon lamp with a white light (400–800 nm) output (10.0 mW/cm$^2$). All groups were in the 5% $O_2$ content solutions.

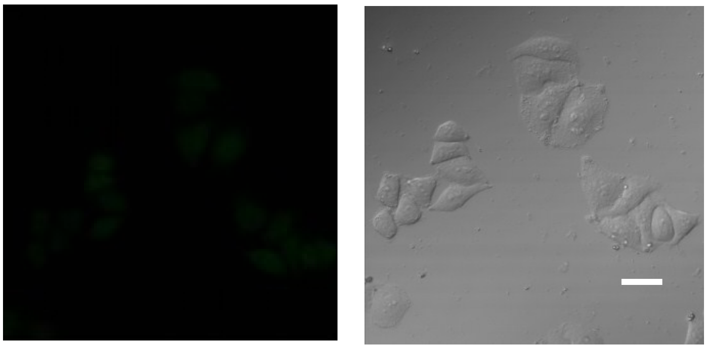

**Figure S12.** Confocal fluorescence images of ROS generation in the Hela cells. Cells were treated with DCFH-DA (10 μM) at 37 °C for 20 min and without light irradiation. Cells were viewed 15 min later in green channel ($\lambda_{ex}$ = 488 nm, $\lambda_{ex}$ = 500–540 nm). All the images share the same scale bar of 30 μm. Images were taken at 25 °C.

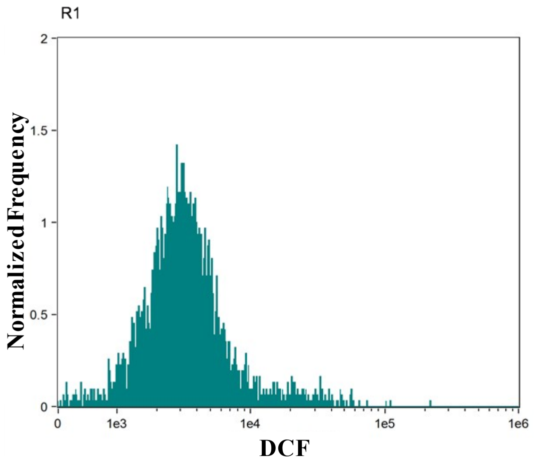


**Figure S13.** Flow cytometric assay of fluorescence intensity of DCF in control group of **Ru1**. Cells were incubated with 5 μM **Ru1** for 2 h and without light irradiation. The concentrations of DCFH-DA were 10 μM and incubation time was 15 min. Cells were measured 30 min later.

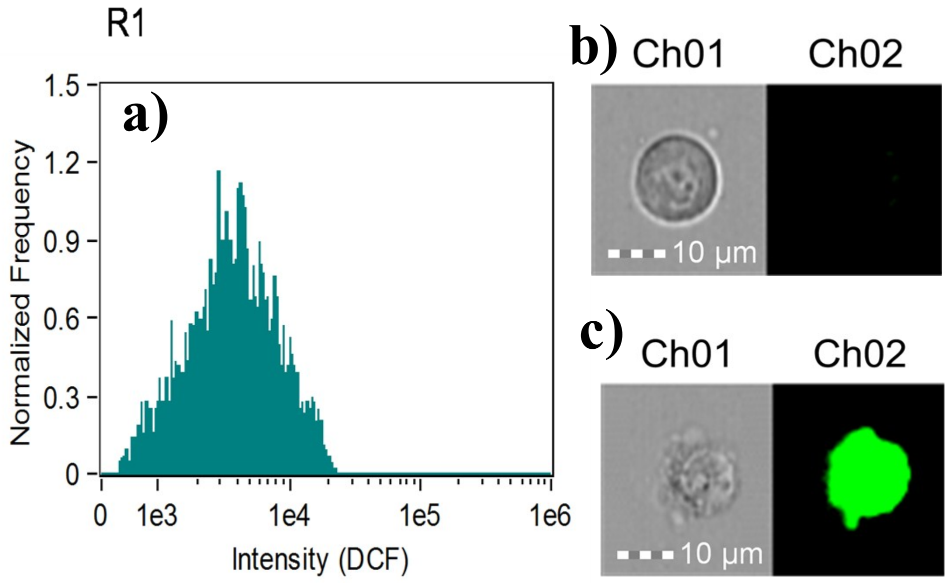


**Figure S14.** a) Flow cytometric assay of fluorescence intensity of DCF in control group of **Ru2**. Fluorescent images of control group (b) and PDT group of **Ru2** (c) were obtained from Flow cytometric assay. Cells of a) were incubated with 5 μM **Ru2** for 2 h and without light irradiation. The concentrations of DCFH-DA were 10 μM and incubation time was 15 min. Cells were measured 30 min later.

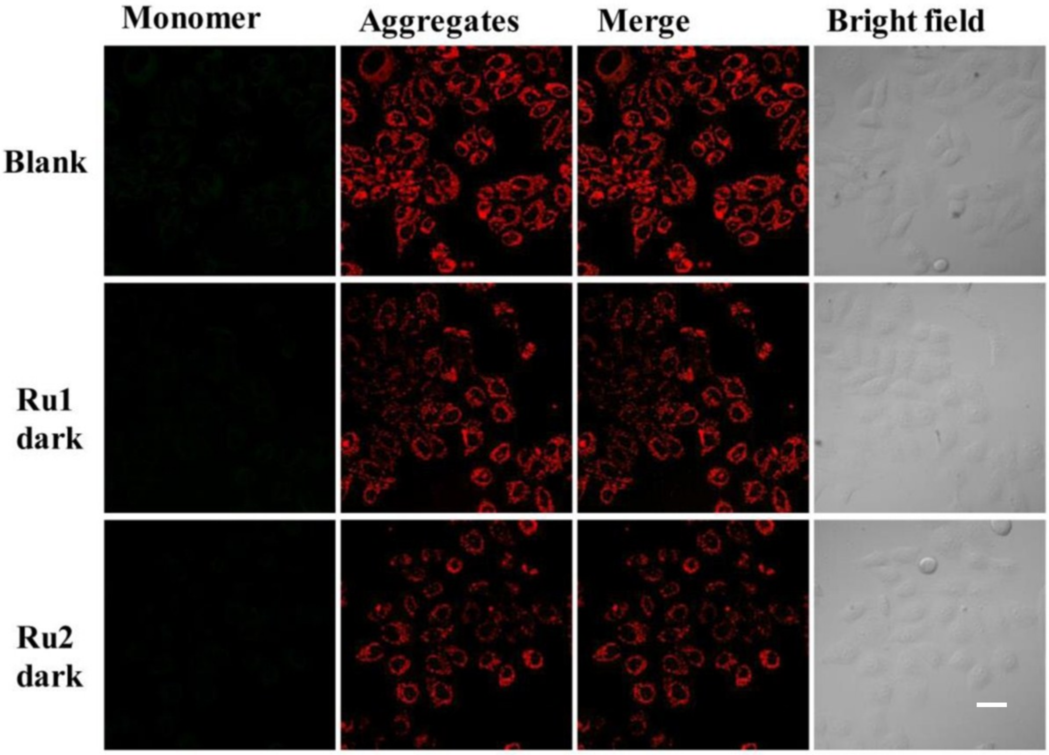


**Figure S15.** Fluorescence images of JC-1 stained Hela cells. Cells were incubated with Ru(II) complexes and protected from light and then stained with JC-1. Cells were viewed in red channel for J-aggregates ($\lambda_{ex}$ = 515 nm, $\lambda_{em}$ = 580–640 nm) and green channel for JC-1 monomer ($\lambda_{ex}$ = 515 nm, $\lambda_{em}$ = 530–560 nm), respectively. All the images share the same scale bar of 50 μm.

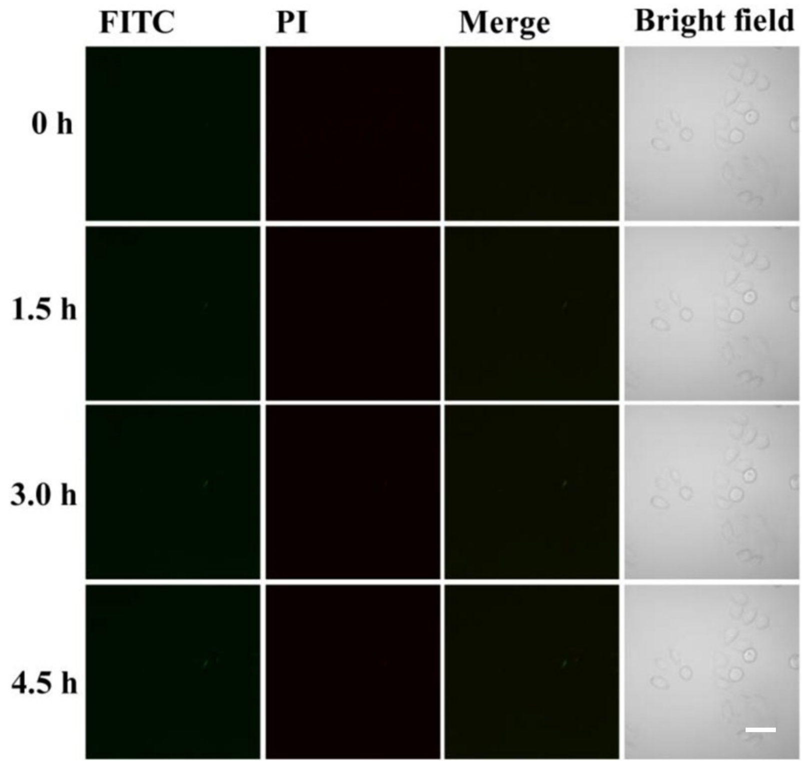


**Figure S16.** Time-lapse fluorescence images of Hela cells. The cells were cultured at 37 °C for 2 h and then stained with Annexin V-FITC/PI under normoxia. Cells were viewed in green channel for Annexin V-FITC ($\lambda_{ex}$ = 488 nm, $\lambda_{em}$ = 500–560 nm) and red channel for PI ($\lambda_{ex}$ = 488 nm, $\lambda_{em}$ = 600–680 nm), respectively. The images were taken at 0–4.5 h with an interval of 1.5 h after the irradiation. All the images share the same scale bar of 50 µm. Images were taken at 25 °C.

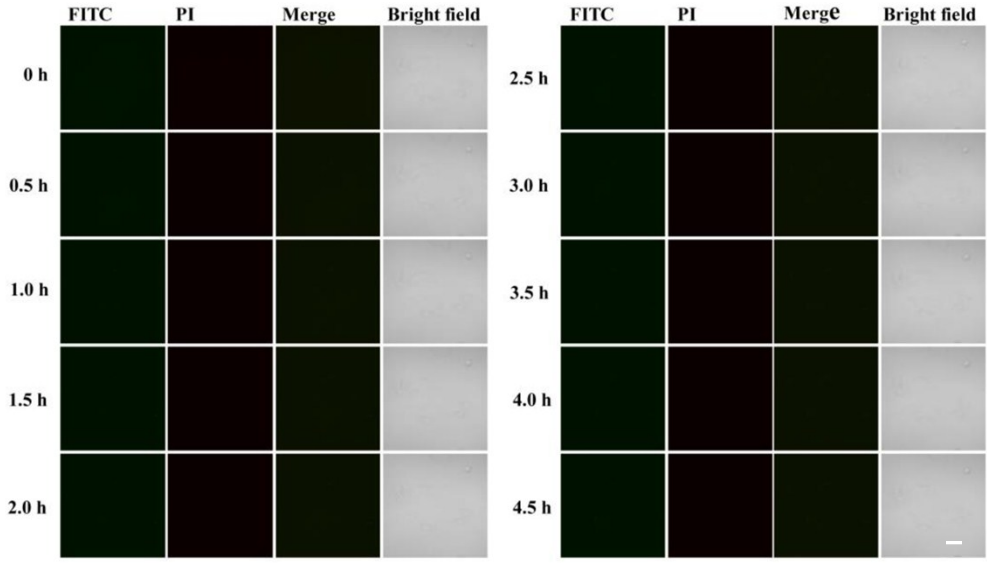


**Figure S17.** Time-lapse fluorescence images of **Ru1** loaded Hela cells. The cells were incubated with **Ru1** (5 μM) at 37 °C for 2 h under dark conditions and then stained with Annexin V-FITC/PI under normoxia. Cells were viewed in green channel for Annexin V-FITC ($\lambda_{ex}$ = 488 nm, $\lambda_{em}$ = 500–560 nm) and red channel for PI ($\lambda_{ex}$ = 488 nm, $\lambda_{em}$ = 600–680 nm), respectively. The images were taken at 0–4.5 h with an interval of 0.5 h after the irradiation. All the images share the same scale bar of 50 μm. Images were taken at 25 °C.

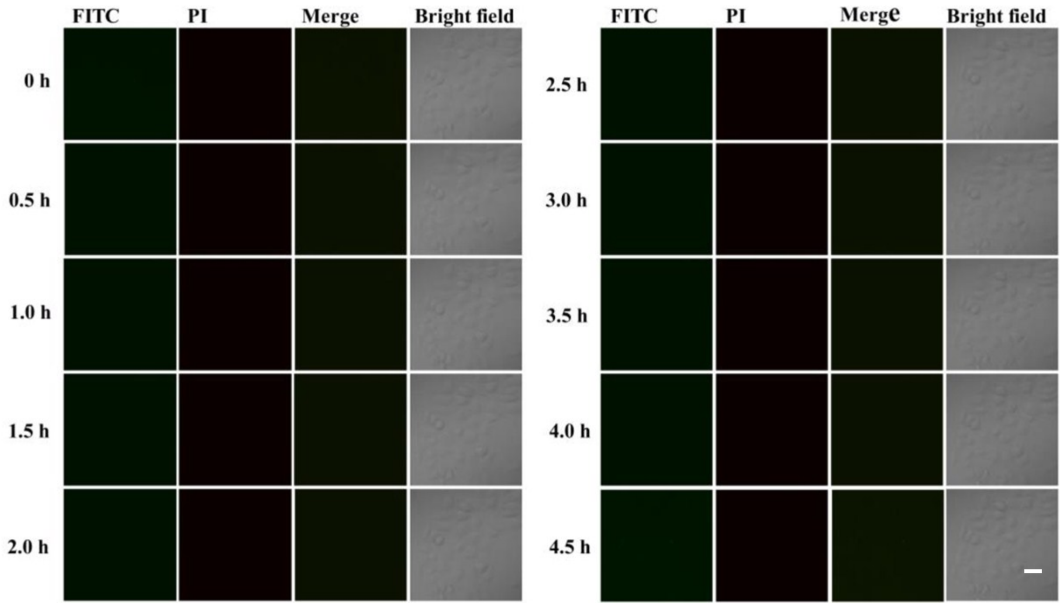


**Figure S18.** Time-lapse fluorescence images of **Ru1** loaded Hela cells after 10 min light (400–800 nm, 35 mW/cm$^2$) irradiation under normoxia. The cells were incubated with **Ru1** (5 µM) at 37 °C for 2 h under normoxia and then stained Annexin V-FITC/PI. Cells were viewed in green channel for Annexin V-FITC ($\lambda_{ex}$ = 488 nm, $\lambda_{em}$ = 500–560 nm) and red channel for PI ($\lambda_{ex}$ = 488 nm, $\lambda_{em}$ = 600–680 nm), respectively. The images were taken at 0–4.5 h with an interval of 0.5 h after the irradiation. All the images share the same scale bar of 50 µm. Images were taken at 25 °C.

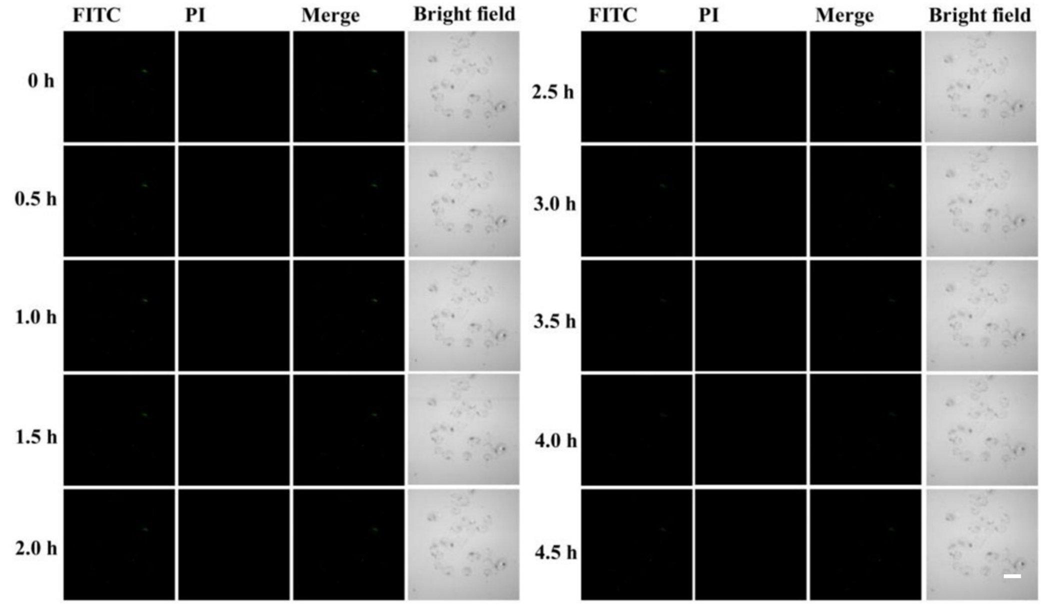


**Figure S19.** Time-lapse fluorescence images of **Ru2** loaded Hela cells. The cells were incubated with **Ru2** (5 μM) at 37 °C for 2 h under dark conditions and then stained with Annexin V-FITC/PI under normoxia. Cells were viewed in green channel for Annexin V-FITC ($\lambda_{ex}$ = 488 nm, $\lambda_{em}$ = 500–560 nm) and red channel for PI ($\lambda_{ex}$ = 488 nm, $\lambda_{em}$ = 600–680 nm), respectively. The images were taken at 0–4.5 h with an interval of 0.5 h after the irradiation. All the images share the same scale bar of 50 μm. Images were taken at 25 °C.

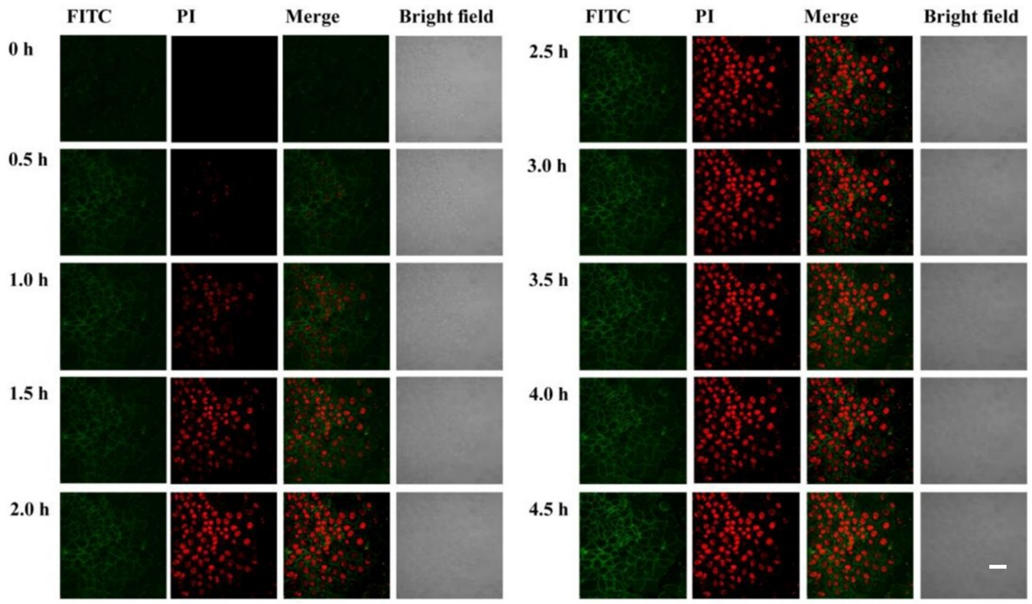


**Figure S20.** Time-lapse fluorescence images of **Ru2** loaded Hela cells after 10 min light (400–800 nm, 35 mW/cm$^2$) irradiation under normoxia. The cells were incubated with **Ru2** (5 μM) at 37 °C for 2 h under normoxia and then stained Annexin V-FITC/PI. Cells were viewed in green channel for Annexin V-FITC ($\lambda_{ex}$ = 488 nm, $\lambda_{em}$ = 500–560 nm) and red channel for PI ($\lambda_{ex}$ = 488 nm, $\lambda_{em}$ = 600–680 nm), respectively. The images were taken at 0–4.5 h with an interval of 0.5 h after the irradiation. All the images share the same scale bar of 50 μm. Images were taken at 25 °C.

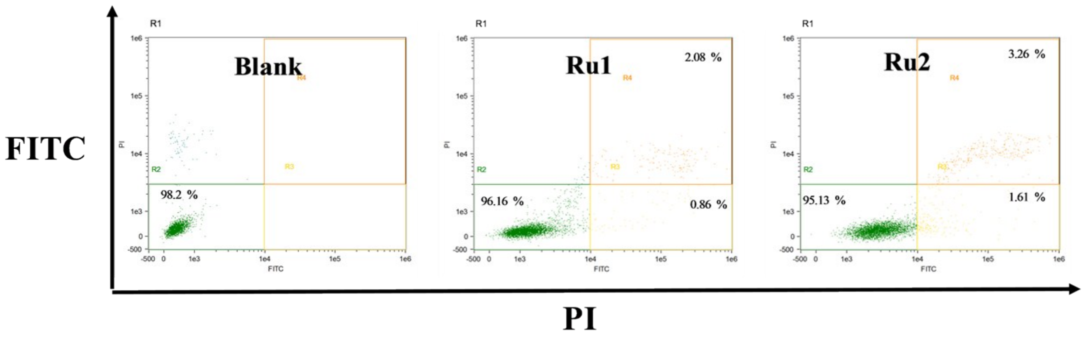


**Figure S21.** Flow cytometry quantification of Annexin V-FITC and PI double labeled HeLa cells. The cells were incubated with 5 μM Ru(II) complexes at 37 °C for 2 h under normoxia and without light irradiation.

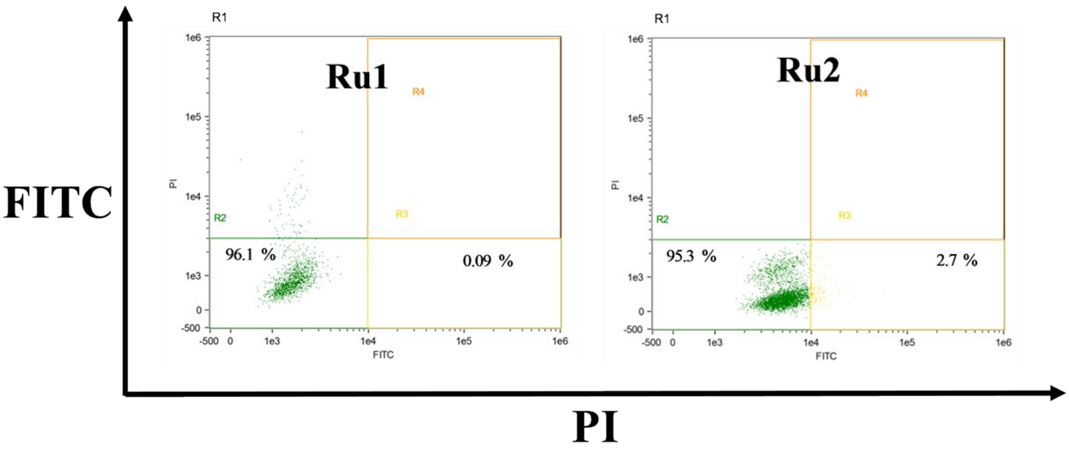


**Figure S22.** Flow cytometry quantification of Annexin V-FITC and PI double labeled HeLa cells. The cells were incubated with 5 μM Ru(II) complex at 37 °C for 2 h under hypoxia and without light irradiation.

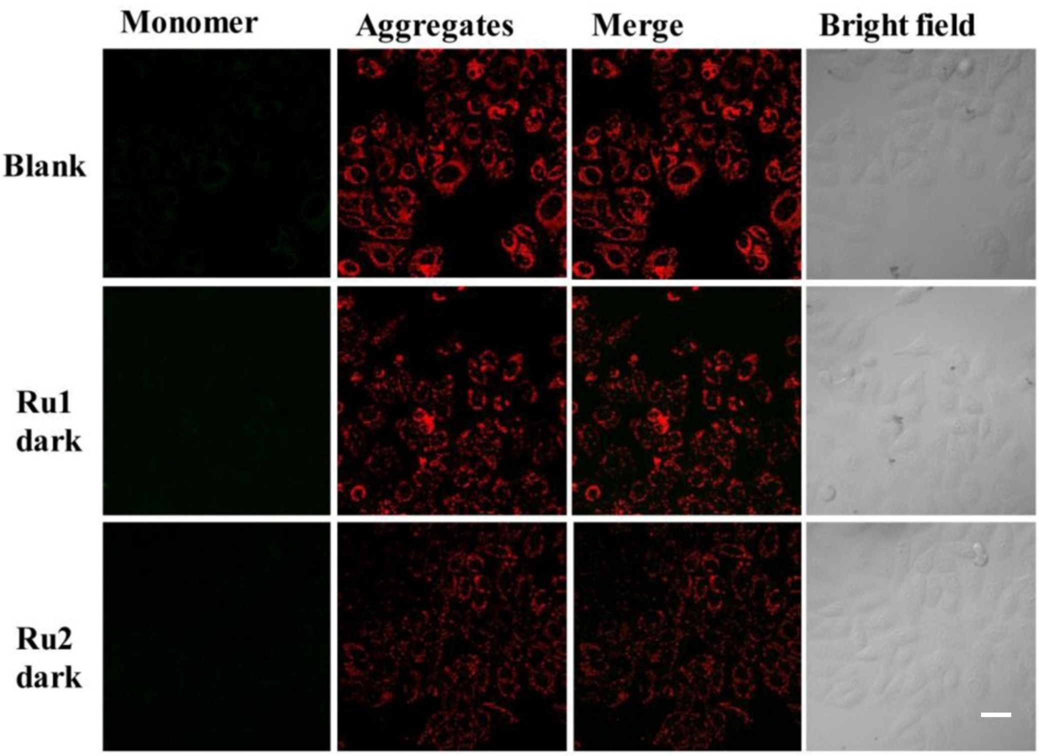


**Figure S23.** Fluorescence images of JC-1 stained Hela cells. Cells were incubated with Ru(II) complexes and protected from light under hypoxia and then stained with JC-1. Cells were viewed in red channel for J-aggregates ($\lambda_{ex}$ = 515 nm, $\lambda_{em}$ = 580–640 nm) and green channel for JC-1 monomer ($\lambda_{ex}$ = 515 nm, $\lambda_{em}$ = 530–560 nm), respectively. All the images share the same scale bar of 50 μm.

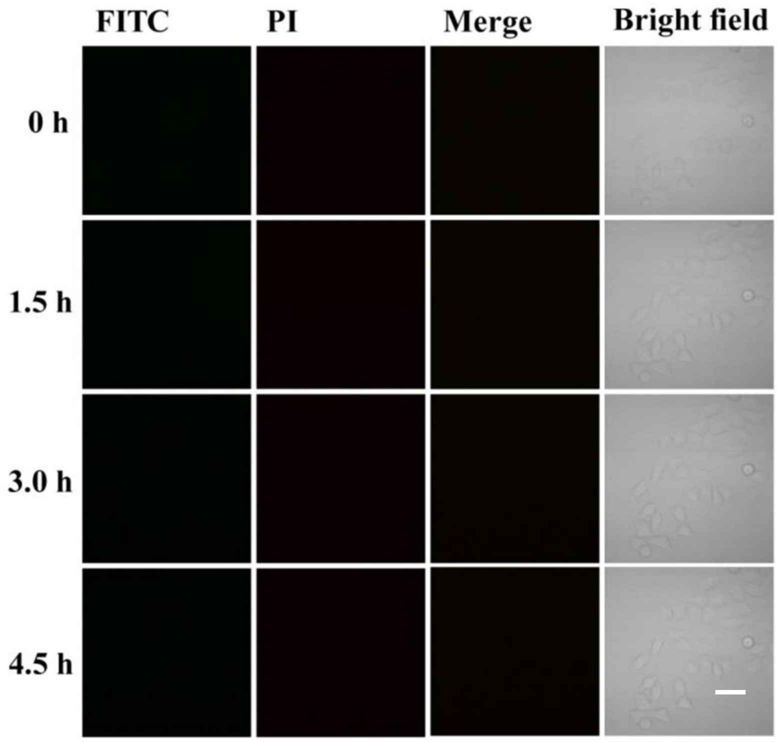


**Figure S24.** Time-lapse fluorescence images of Hela cells under hypoxia. The cells were cultured at 37 °C for 2 h under hypoxia and then stained with Annexin V-FITC/PI. Cells were viewed in green channel for Annexin V-FITC ($\lambda_{ex}$ = 488 nm, $\lambda_{em}$ = 500–560 nm) and red channel for PI ($\lambda_{ex}$ = 488 nm, $\lambda_{em}$ = 600–680 nm), respectively. The images were taken at 0–4.5 h with an interval of 1.5 h after the irradiation. All the images share the same scale bar of 50 μm. Images were taken at 25 °C.

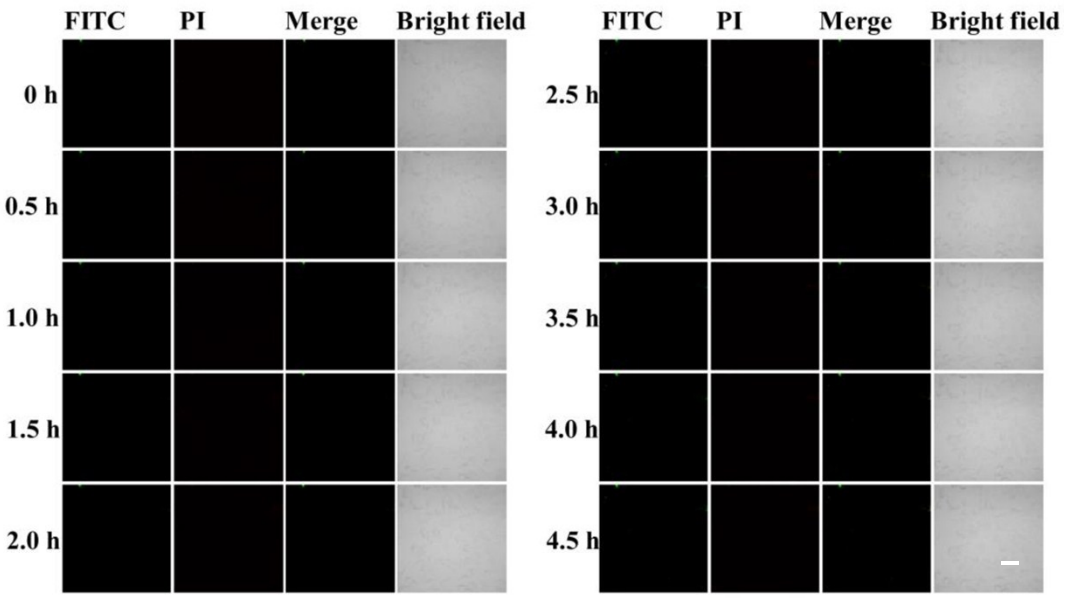


**Figure S25.** Time-lapse fluorescence images of **Ru1** loaded Hela cells under hypoxia. The cells were incubated with **Ru1** (5 μM) at 37 °C for 2 h under hypoxia (5 % $O_2$) and then stained with Annexin V-FITC/PI and protected from light. Cells were viewed in green channel for Annexin V-FITC ($\lambda_{ex}$ = 488 nm, $\lambda_{em}$ = 500–560 nm) and red channel for PI ($\lambda_{ex}$ = 488 nm, $\lambda_{em}$ = 600–680 nm), respectively. The images were taken at 0–4.5 h with an interval of 0.5 h after the irradiation. All the images share the same scale bar of 50 μm. Images were taken at 25 °C.

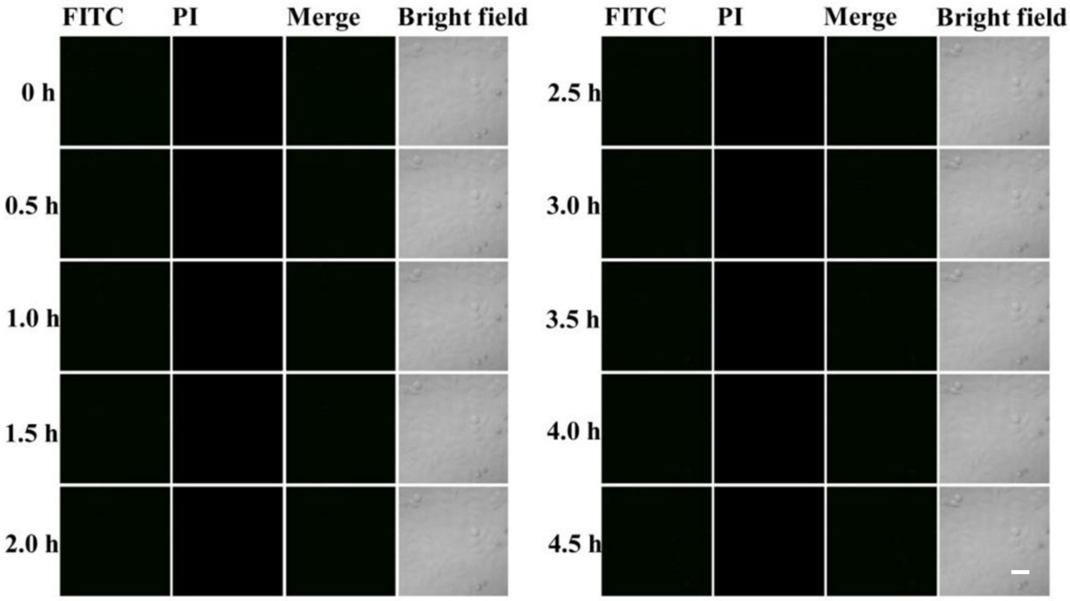


**Figure S26.** Time-lapse fluorescence images of **Ru2** loaded Hela cells under hypoxia. The cells were incubated with **Ru2** (5 µM) at 37 °C for 2 h under hypoxia (5 % $O_2$) and then stained with Annexin V-FITC/PI and protected from light. Cells were viewed in green channel for Annexin V-FITC ($\lambda_{ex}$ = 488 nm, $\lambda_{em}$ = 500–560 nm) and red channel for PI ($\lambda_{ex}$ = 488 nm, $\lambda_{em}$ = 600–680 nm), respectively. The images were taken at 0–4.5 h with an interval of 0.5 h after the irradiation. All the images share the same scale bar of 50 µm. Images were taken at 25 °C.

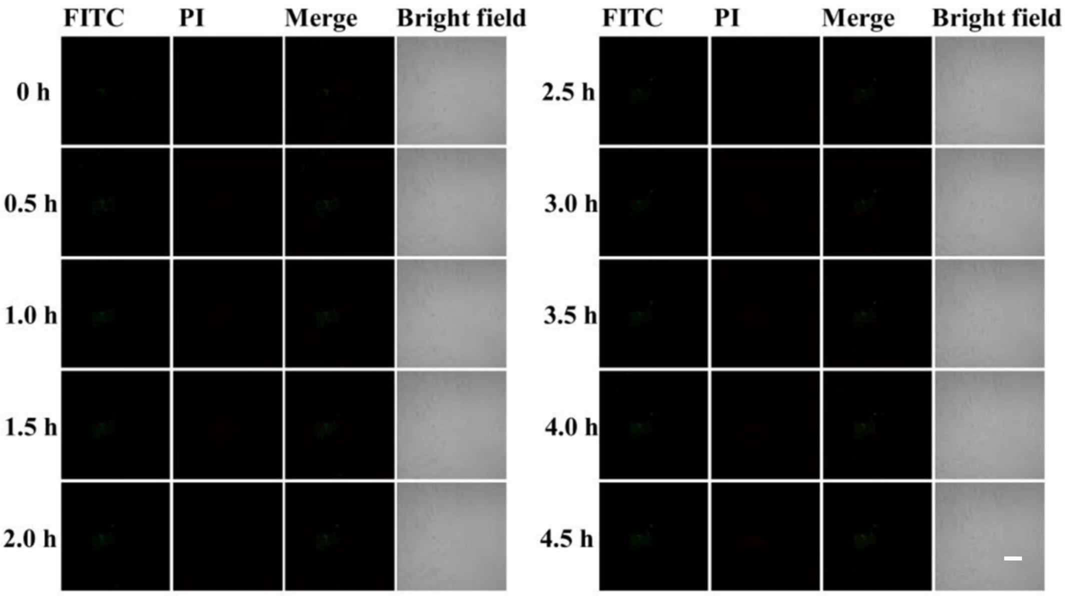


**Figure S27.** Time-lapse fluorescence images of **Ru1** loaded Hela cells after 10 min light (400–800 nm, 35 mW/cm$^2$) irradiation under hypoxia. The cells were incubated with **Ru1** (5 μM) at 37 °C for 2 h under hypoxia (5 % $O_2$) and then stained with Annexin V-FITC/PI. Cells were viewed in green channel for Annexin V-FITC ($\lambda_{ex}$ = 488 nm, $\lambda_{em}$ = 500–560 nm) and red channel for PI ($\lambda_{ex}$ = 488 nm, $\lambda_{em}$ = 600–680 nm), respectively. The images were taken at 0-4.5 h with an interval of 0.5 h after the irradiation. All the images share the same scale bar of 50 μm. Images were taken at 25 °C.

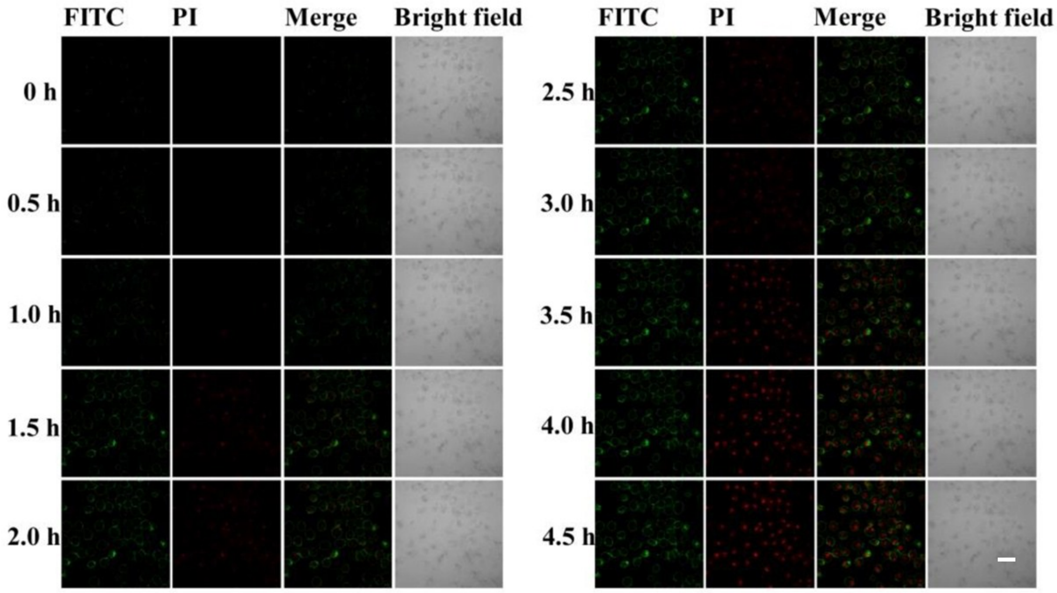


**Figure S28.** Time-lapse fluorescence images of **Ru2** loaded Hela cells after 10 min light (400–800 nm, 35 mW/cm$^2$) irradiation under hypoxia. The cells were incubated with **Ru2** (5 μM) at 37 °C for 2 h under hypoxia (5 % $O_2$) and then stained with Annexin V-FITC/PI. Cells were viewed in green channel for Annexin V-FITC ($\lambda_{ex}$ = 488 nm, $\lambda_{em}$ = 500–560 nm) and red channel for PI ($\lambda_{ex}$ = 488 nm, $\lambda_{em}$ = 600–680 nm), respectively. The images were taken at 0–4.5 h with an interval of 0.5 h after the irradiation. All the images share the same scale bar of 50 μm. Images were taken at 25 °C.

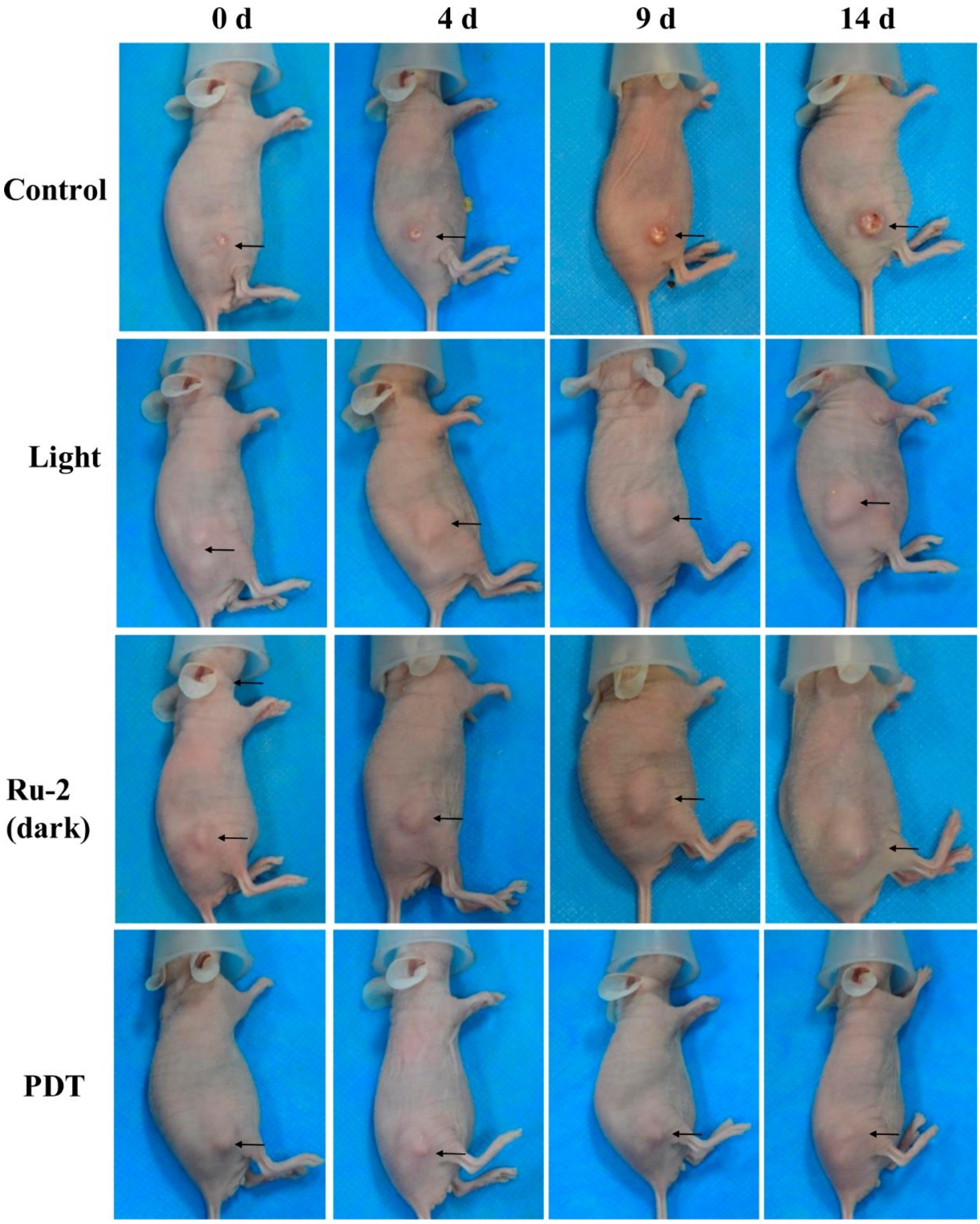


**Figure S29.** Photographs of mice in different groups, the tumor bearing mice were treated with PBS (Control group), light irradiation only, **Ru2** without irradiation and PDT, respectively.

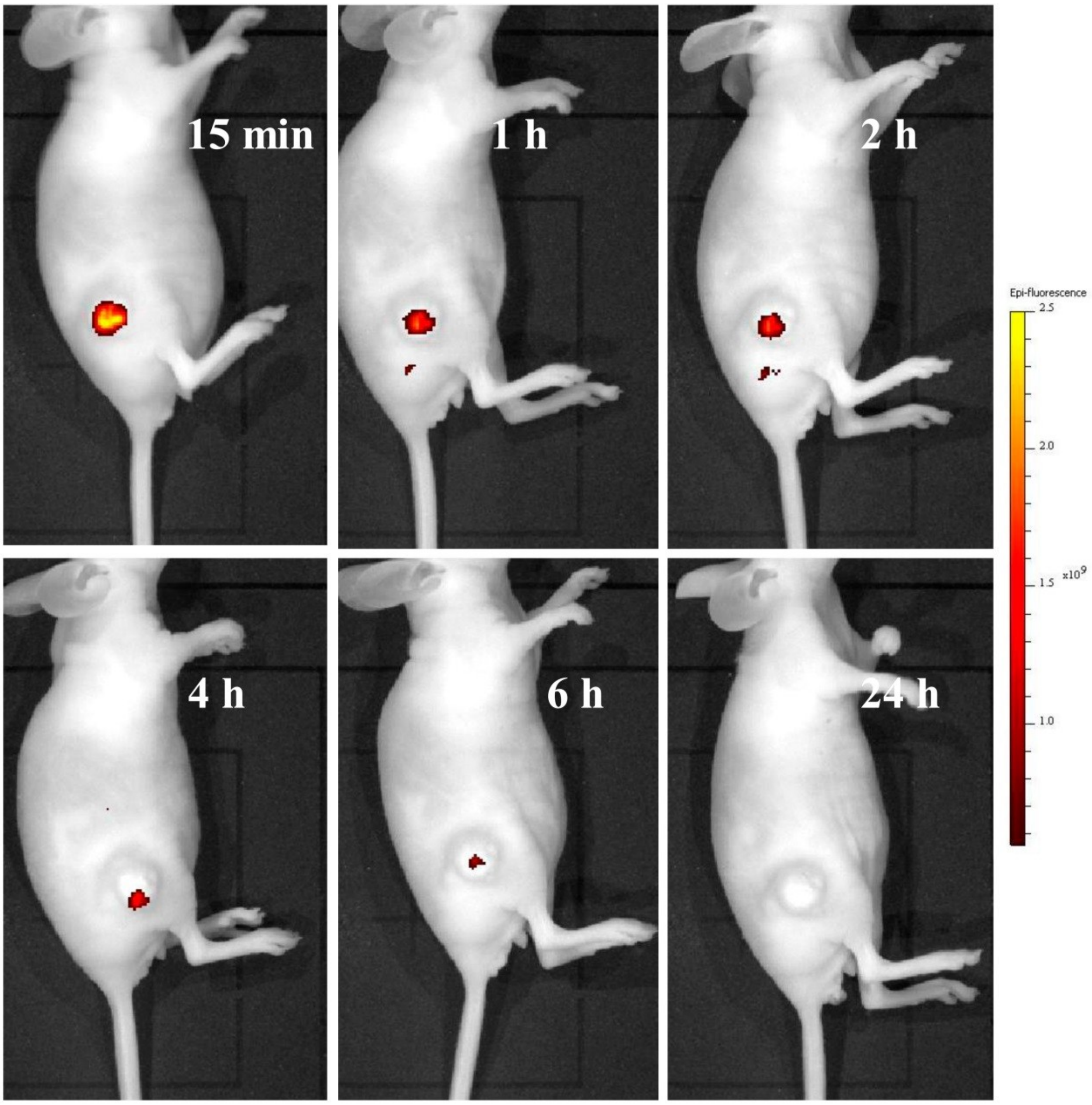


**Figure S30.** In vivo fluorescence imaging of tumor bearing mice at 15 min, 1 h, 2 h, 4 h, 6 h and 24 h post injection of $Ru(bpy)_3(PF_6)_2$. Scale bar is 1 cm.

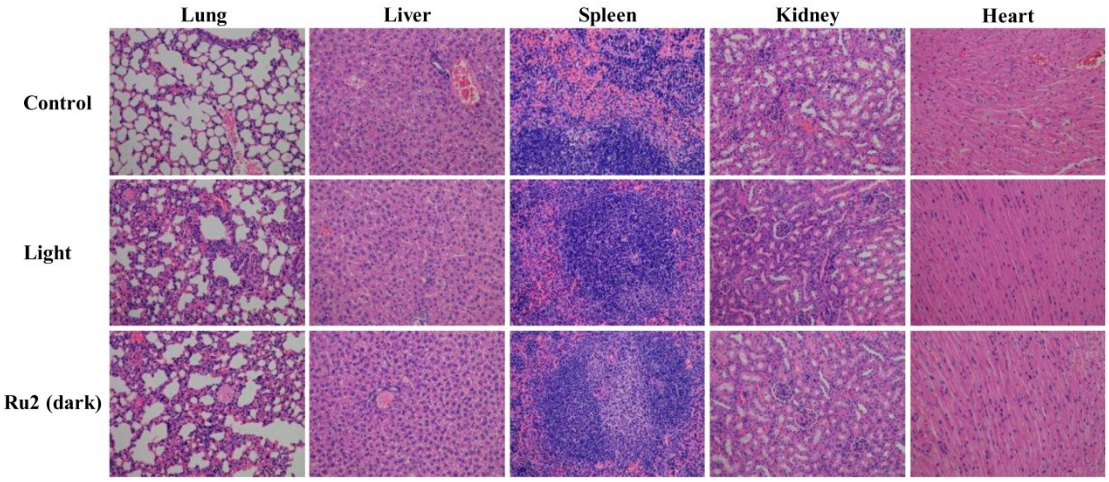


**Figure S31.** H&E stained tissue slices of normal organs (Lung, liver, spleen, kidney, heart) in different groups after 14 days treatment. The images share the same scale bar of 100 μm.

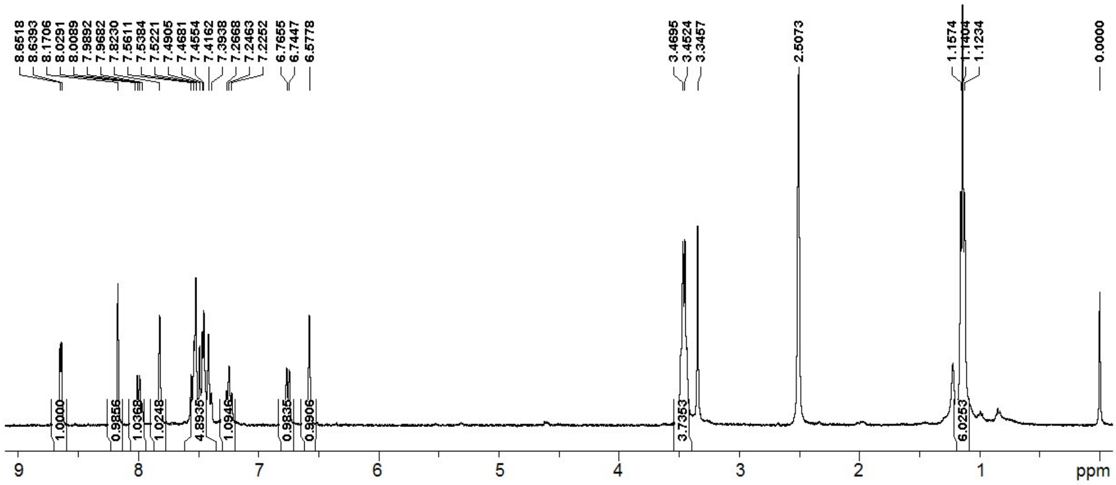

**Figure S32.** $^{1}$H NMR spectrum of compound **4** in DMSO-$d_6$.

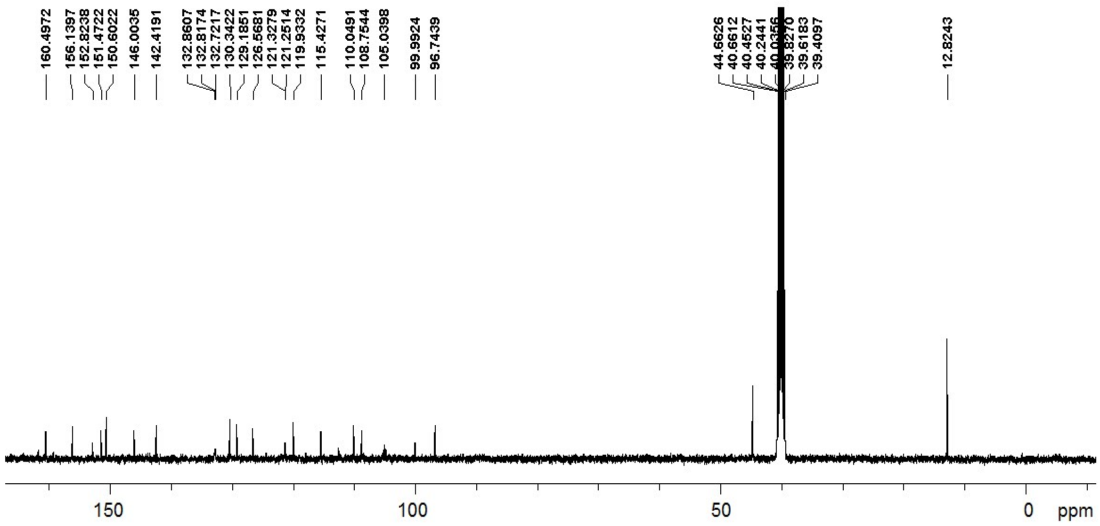


**Figure S33.** $^{13}C$ NMR spectrum of compound **4** in DMSO-$d_6$.

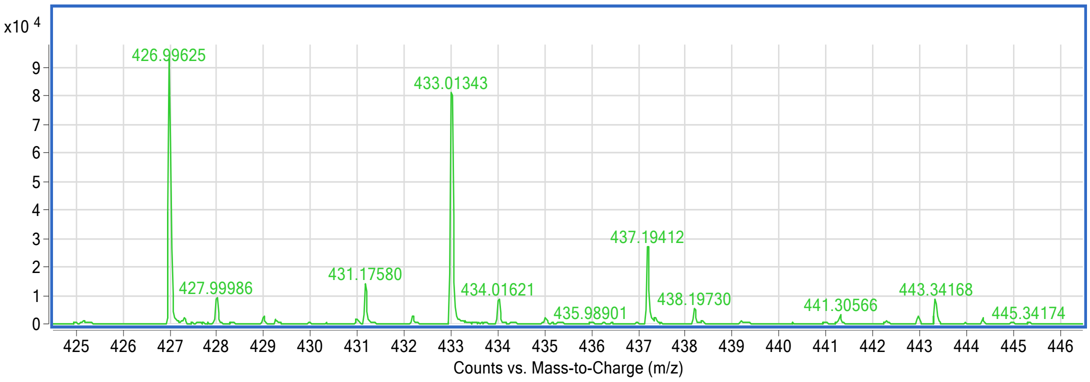


**Figure S34.** MS spectrum of compound **4**.

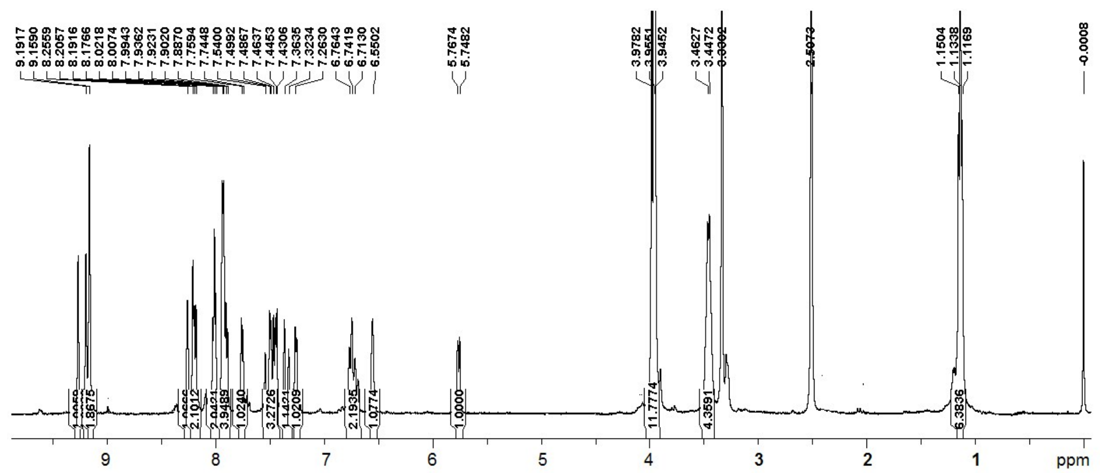

**Figure S35.** $^1$H NMR spectrum of **Ru2** in DMSO-$d_6$.

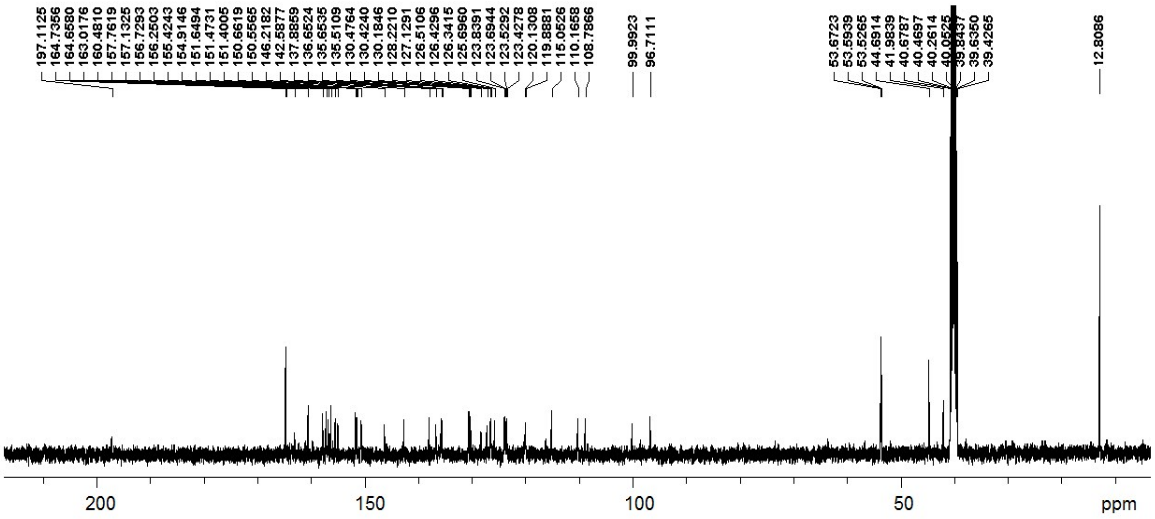

**Figure S36.** $^{13}C$ NMR spectrum of **Ru2** in DMSO-$d_6$.

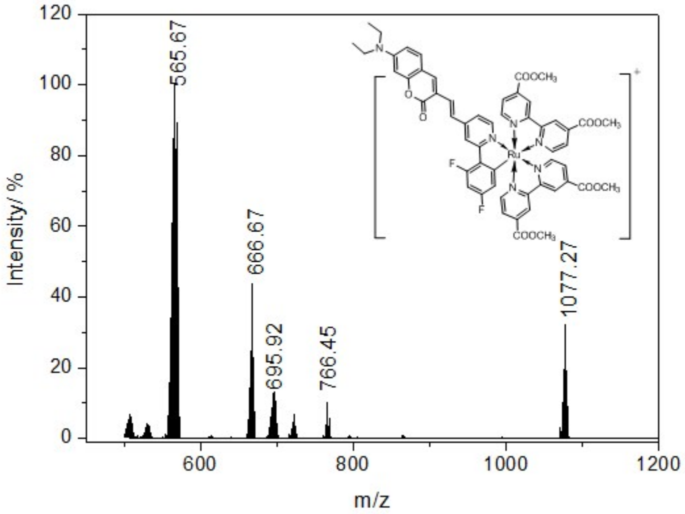


**Figure S37.** MS spectrum of **Ru2**.

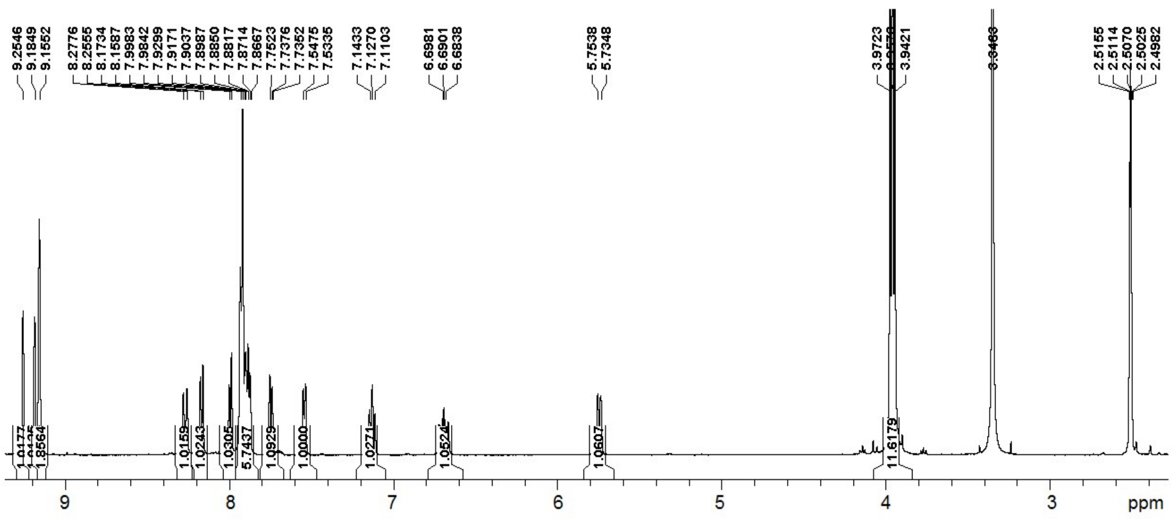

**Figure S38.** $^{1}H$ NMR spectrum of **Ru1** in DMSO-$d_6$.

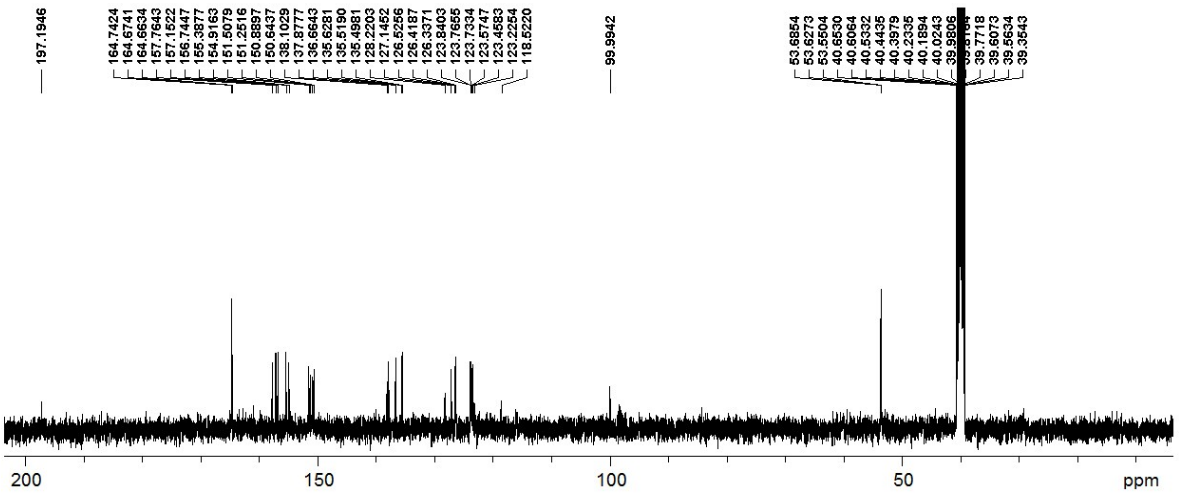


**Figure S39.** $^{13}C$ NMR spectrum of **Ru1** in DMSO-$d_6$.

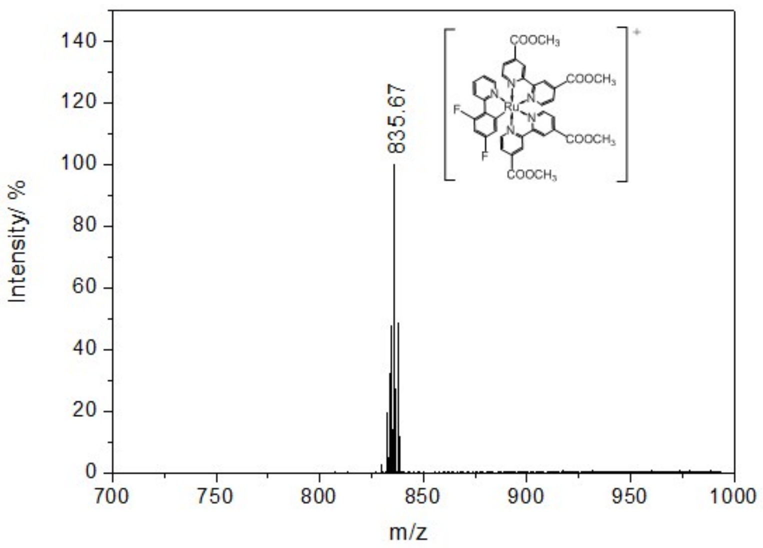


**Figure S40.** MS spectrum of **Ru1**.
